# Cryo-EM of Aβ fibrils from mouse models find tg-APP_ArcSwe_ fibrils resemble those found in patients with sporadic Alzheimer’s disease

**DOI:** 10.1038/s41593-023-01484-4

**Published:** 2023-11-16

**Authors:** Mara Zielinski, Fernanda S. Peralta Reyes, Lothar Gremer, Sarah Schemmert, Benedikt Frieg, Luisa U. Schäfer, Antje Willuweit, Lili Donner, Margitta Elvers, Lars N. G. Nilsson, Stina Syvänen, Dag Sehlin, Martin Ingelsson, Dieter Willbold, Gunnar F. Schröder

**Affiliations:** 1 Institute of Biological Information Processing, Structural Biochemistry (IBI-7), Forschungszentrum Jülich, Jülich, Germany; 2 JuStruct, Jülich Center for Structural Biology, Forschungszentrum Jülich, Jülich, Germany; 3 Institut für Physikalische Biologie, Heinrich Heine University Düsseldorf, Düsseldorf, Germany; 4 Institute of Neuroscience and Medicine, Medical Imaging Physics (INM-4), Forschungszentrum Jülich, Jülich, Germany; 5 Department of Vascular and Endovascular Surgery, University Hospital Düsseldorf, Heinrich Heine University, Düsseldorf, Germany; 6 Department of Pharmacology, Institute of Clinical Medicine, University of Oslo and Oslo University Hospital, Oslo, Norway; 7 Department of Public Health and Caring Sciences, Molecular Geriatrics, Rudbeck Laboratory, Uppsala University, Uppsala, Sweden; 8 Krembil Brain Institute, University Health Network, Toronto, Ontario Canada; 9 Tanz Centre for Research in Neurodegenerative Diseases, Departments of Medicine and Laboratory Medicine & Pathobiology, University of Toronto, Toronto, Ontario Canada; 10 Physics Department, Heinrich Heine University Düsseldorf, Düsseldorf, Germany; 11 Present Address: Life and Medical Sciences (LIMES) Institute, University of Bonn, Bonn, Germany

**Keywords:** Molecular neuroscience, Cryoelectron microscopy

## Abstract

The use of transgenic mice displaying amyloid-β (Aβ) brain pathology has been essential for the preclinical assessment of new treatment strategies for Alzheimer’s disease. However, the properties of Aβ in such mice have not been systematically compared to Aβ in the brains of patients with Alzheimer’s disease. Here, we determined the structures of nine ex vivo Aβ fibrils from six different mouse models by cryogenic-electron microscopy. We found novel Aβ fibril structures in the APP/PS1, ARTE10 and tg-SwDI models, whereas the human type II filament fold was found in the ARTE10, tg-APP_Swe_ and APP23 models. The tg-APP_ArcSwe_ mice showed an Aβ fibril whose structure resembles the human type I filament found in patients with sporadic Alzheimer’s disease. A detailed assessment of the Aβ fibril structure is key to the selection of adequate mouse models for the preclinical development of novel plaque-targeting therapeutics and positron emission tomography imaging tracers in Alzheimer’s disease.

## Abstract (web summary)

This study by Zielinski et al. used cryo-EM to compare Aβ fibril structures from mouse models to those from patients with Alzheimer’s disease. It revealed that tg-APP_ArcSwe_ mice exhibit fibrils resembling those predominantly found in sporadic Alzheimer’s disease cases.

## Main

Alzheimer’s disease is the most common form of dementia and is neuropathologically defined by the presence of extracellular plaques containing Aβ in the brain parenchyma and intraneuronal neurofibrillary tangles containing phosphorylated tau^1–4^. In the amyloidogenic pathway, Aβ is sequentially cleaved from the amyloid precursor protein (APP) by β-secretases and ɣ-secretases^5,6^. Peptides between 37 and 43 residues in length are generated, with those of 40 (Aβ40) and 42 (Aβ42) residues in length being the most abundant^7^. These Aβ monomers tend to aggregate into insoluble fibrils, the structure of which has been extensively studied in vitro by cryogenic-electron microscopy (cryo-EM) and solid-state nuclear magnetic resonance spectroscopy, revealing a spectrum of different polymorphs^8–14^. However, these fibrils are structurally different from both Aβ40 and Aβ42 fibrils derived by seeded growth from the brain tissue of patients with Alzheimer’s disease^15,16^ as well as Aβ40 and Aβ42 fibrils extracted from the meninges^17^ and parenchyma^18^, respectively, of patients with Alzheimer’s disease. Two human fibril polymorphs have been determined: ‘type I filaments’, which are mostly associated with sporadic Alzheimer’s disease (SAD), and ‘type II filaments’, which are observed in familial Alzheimer’s disease (FAD) and other neurodegenerative disorders with amyloid brain pathology^18^. Animal models are an important tool to study the pathogenesis of Alzheimer’s disease and to conduct preclinical testing of novel therapeutics^19^. Commonly used animal models are transgenic mice that mimic different clinical characteristics of the disease, such as the accumulation of Aβ by overexpressing human APP^20^. Although a new generation of mouse models, so-called knock-in mice, has been developed to circumvent problems associated with the overexpression of APP, these models also have shortcomings, such as an artificially high Aβ42:Aβ40 ratio. The structures of Aβ fibrils extracted from the knock-in APP^NL-G-F^ and the knock-in APP^NL-F^ mouse models were recently determined by cryo-EM^18,21,22^. While the APP^NL-F^ Aβ42 fibril resembles the human type II Aβ42 fold, the fold of the APP^NL-G-F^ Aβ42(E22G) fibrils differs from those extracted from human brain tissue.

Aggregated Aβ is a common target for drug development^23^, and the first two disease-modifying treatments, based on immunotherapy with the anti-Aβ antibodies aducanumab and lecanemab, have now received accelerated approval by the US Food and Drug Administration. Lecanemab was recently shown to be effective in removing plaques and partially normalizing blood and cerebrospinal fluid biomarkers, and it also showed a modest clinical effect^24^. Its murine parent antibody, mAb158, was developed primarily against oligomers and protofibrils, intermediately sized soluble Aβ aggregates^25,26^. The structure of these Aβ aggregates remains elusive, but a recent study^27^ showed interactions between lecanemab and Aβ fibrils that were present in ‘ultracentrifugal supernatants of aqueous extracts from Alzheimer’s disease brains’. In spite of the recent success, developing novel drugs for Alzheimer’s disease has overall been challenging, with a drug development failure rate of almost 100%^28^. Structural differences in human and murine Aβ fibrils might help to improve our understanding of why fibril-targeting drug candidates show efficacy when tested in mouse models but then fail to show the desired effect in clinical trials^20,29–34^. Additionally, structural differences between Aβ polymorphs potentially influence the availability of binding sites for positron emission tomography (PET) imaging tracers, resulting in some plaque pathologies not being detected by PET tracers^35^. Elucidating the structures of Aβ fibrils in humans and in mice could therefore support the development of new tracers that are targeted towards specific fibril structures and plaque pathologies.

**Fig. 1:**
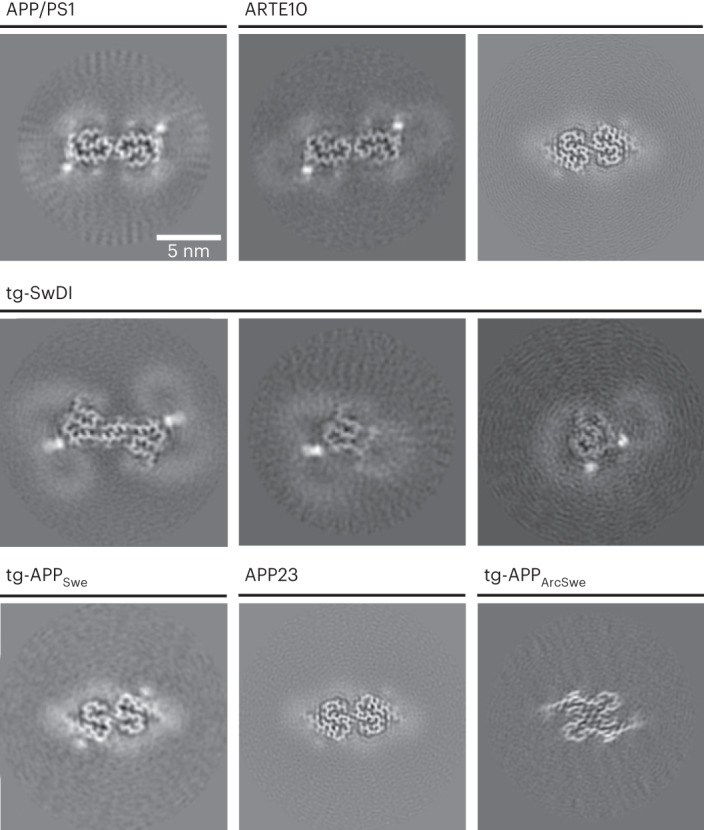
Cryo-EM reconstructions of Aβ fibrils extracted from APP/PS1, ARTE10, tg-SwDI, tg-APP_Swe_, APP23 and tg-APP_ArcSwe_ mouse brain tissue. For every reconstructed fibril, a projection of the reconstructed density including approximately one β-rung is shown. The scale bar in the top left panel applies to all shown panels. From upper left to lower right: murine type III (APP/PS1), murine type III (ARTE10), murine type II (ARTE10), DI1, DI2, DI3, murine type II (tg-APP_Swe_), murine type II (APP23) and murine_Arc_ type I. The number of fibril segments used to generate the reconstructions is given in Extended Data Table 2.

**Extended Data Fig. 1:**
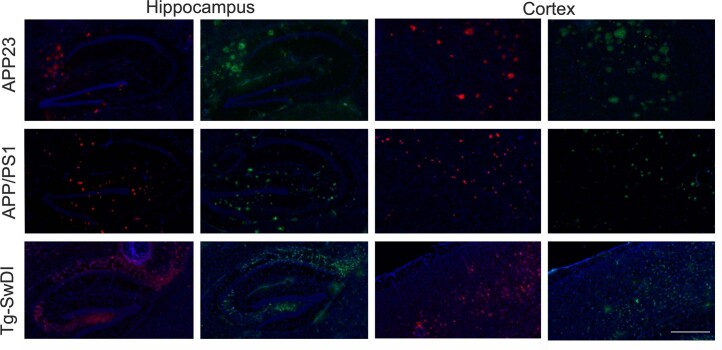
Immunohistochemical characterisation of mouse brain tissue. Immunohistochemical staining showing Aβ plaques in the hippocampus (left images) and cortex (right images) of APP23 (upper panel), APP/PS1 (middle panel) and tg-SwDI mice (lower panel). Two different stainings were conducted (6E10 labelled in red and 4G8 labelled in green). Nuclear staining was done with DAPI (blue). Representative images are shown (n = 3). Scale bar= 500 μm.

**Extended Data Fig. 2:**
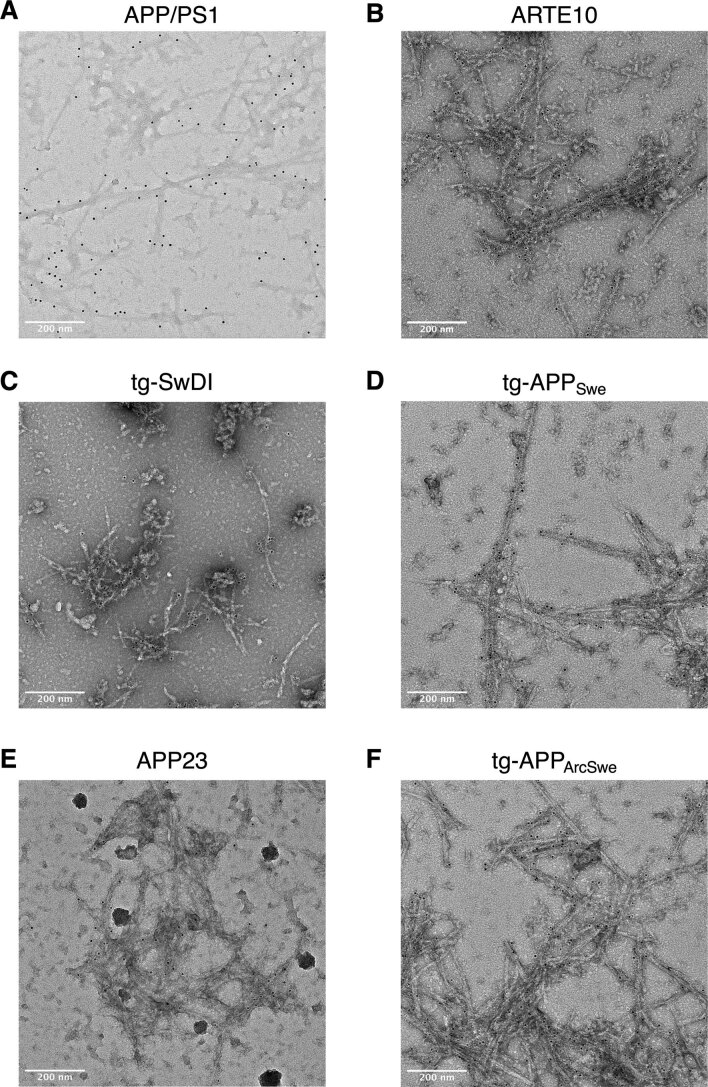
Immunogold negative stain electron microscopy images of purified Aβ fibrils. Immunogold negative-stain electron microscopy images of the purified Aβ fibrils from (A) APP/PS1 (n = 30), (B) ARTE10 (n = 6), (C) tg-SwDI (n = 21), (D) tg-APP_Swe_ (n = 10), (E) APP23 (n = 8) and (F) tg-APP_ArcSwe_ (n = 15) mouse models. NAB228 was used as primary antibody. A goat anti-mouse gold-conjugated antibody with a gold particle diameter of 6 nm was used as secondary antibody.

**Extended Data Fig. 3:**
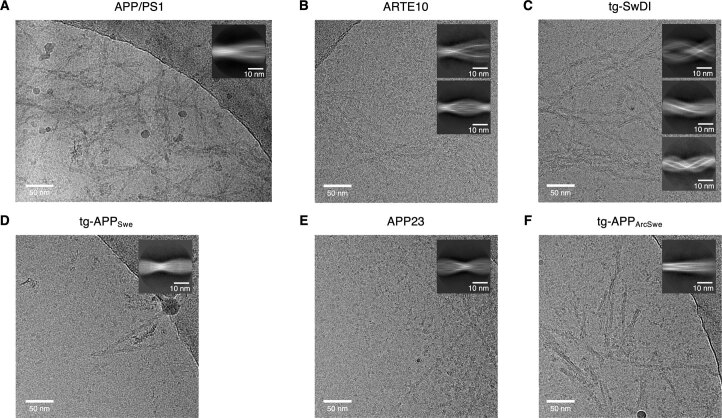
Cryo-EM micrographs and 2D classes of purified Aβ fibrils. One exemplary cryo-EM micrograph and 2D class averages of (A) APP/PS1, (B) ARTE10, (C) tg-SwDI, (D) tg-APP_Swe_, (E) APP23 and (F) tg-APP_ArcSwe_. The number of micrographs recorded for each sample is given in Extended Data Table 2. The number of fibril segments used to generate the displayed 2D classes are given in Extended Data Table 2 (n=final particle images).

Despite the frequent use of transgenic mice in the development of anti-Aβ therapeutics and PET-based diagnostics, the structures of murine Aβ fibrils have not been thoroughly investigated. Here, we extracted Aβ fibrils from the brains of six commonly used mouse models (APP/PS1, ARTE10, tg-SwDI, tg-APP_Swe_, APP23 and tg-APP_ArcSwe_) with a previously described sarkosyl extraction method^18^ and determined their structure by cryo-EM (Fig. 1, Extended Data Figs. 1–3 and Extended Data Tables 1 and 2). The various murine Aβ fibril structures were then compared to the previously reported structures of brain-derived Aβ fibrils from patients with Alzheimer’s disease.

## Results

### Murine type III Aβ fibrils from APP/PS1 and ARTE10 mice

**Fig. 2:**
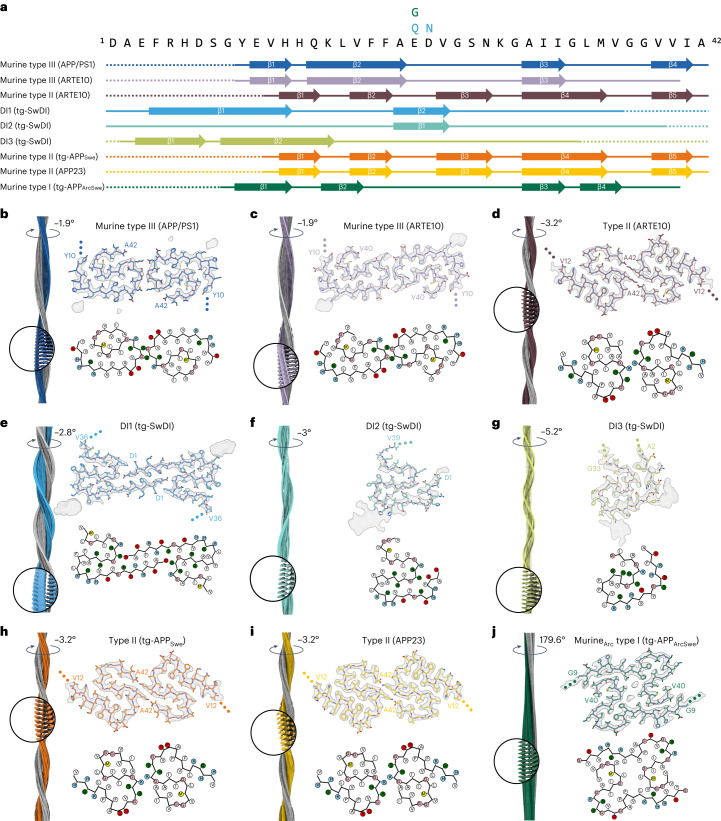
Overview of all murine Aβ fibril structures. **a**, Amino acid sequence of Aβ42. The sequence contains the following mutations for tg-SwDI: E22Q and D23N; and for tg-APP_ArcSwe_: E22G. Solid lines indicate the part of the sequence for which atomic model building was possible (accordingly, dotted lines represent parts of the sequence that were not modeled). Arrows indicate β-strands. **b**–**j**, Each panel shows the reconstructed cryo-EM density along the helical axis with a close-up and a label denoting the helical twist (left); the cryo-EM density map (in transparent gray) with the corresponding atomic model (top right); a schematic of the fold, produced with atom2svg.py^60^ (red, acidic side chain; blue, basic side chain; green, hydrophilic side chain; white, hydrophobic side chain; pink, glycine; yellow, sulfur-containing) (bottom right). Cryo-EM structure of murine type III Aβ42 fibrils from APP/PS1 mouse brain (**b**), murine type III Aβ fibrils from ARTE10 mouse brain (**c**), type II Aβ42 fibrils from ARTE10 mouse brain (**d**), DI1 Aβ fibrils from tg-SwDI mouse brain (**e**), DI2 Aβ fibrils from tg-SwDI mouse brain (**f**), DI3 Aβ fibrils from tg-SwDI mouse brain (**g**), type II Aβ42 fibrils from tg-APP_Swe_ mouse brain (**h**), type II Aβ42 fibrils from APP23 mouse brain (**i**) and murine_Arc_ type I fibrils from tg-APP_ArcSwe_ mouse brain (**j**). For **f** and **g** (DI2 and DI3 fibrils from tg-SwDI mouse brain), the displayed atomic models have limited accuracy owing to the medium resolution.

For the APP/PS1 mice, we observed only one fibril type made of two identical LS-shaped protofilaments related by a C2 symmetry (Figs. 1 and 2a,b). This fibril type, which we call the murine type III Aβ fold, was also found in ARTE10 mouse brain (Fig. 2c) and accounts for 4% of all reconstructed ARTE10 fibrils (Supplementary Table 1). Murine type III fibrils were determined to a resolution of 3.5 Å and 3.3 Å for APP/PS1 and ARTE10 mice, respectively (Extended Data Table 2 and Supplementary Fig. 1a,b). For APP/PS1 murine type III Aβ42 fibrils, atomic model building was possible for the ordered core from residues G9–A42 (Fig. 2a,b). The reconstructed density of ARTE10 murine type III shows only weak carboxy-terminal density; accordingly, an atomic model was built from residues G9–V40 (Fig. 2a,c). The amino-terminal L-turn involves residues Y10–F19 and is mainly stabilized by one hydrophobic cluster composed of Y10, V12, L17, F10, L34 and V36 (Fig. 2b,c and Supplementary Fig. 2a,b). The S-turn, which involves residues F20–V40/A42, is stabilized by two hydrophobic clusters: in the first half of the S-turn between F20 and K28 involving A21, V24 and I31, and the C-terminal second half of the S-turn involving A30, I32, M35, V40 (and A42). The protofilament interface involving residues D23–K28 of murine type III fibrils is stabilized by symmetric salt bridges between D23 and K28 of the opposing subunits.

**Fig. 3:**
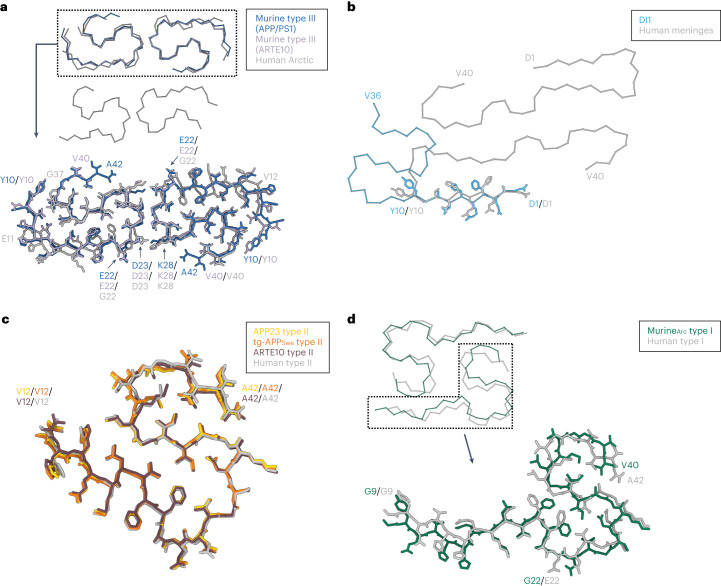
Comparison of brain-derived murine Aβ fibrils to brain-derived human extracted Aβ fibrils. **a**, Comparison of murine type III Aβ fibrils (blue, APP/PS1; lavender, ARTE10) with the cryo-EM structure of human brain-extracted Aß filaments with the E693G (E22G) mutation (gray; PDB 8BG0). **b**, Comparison of the DI1 Aβ fibril (light blue) with the cryo-EM structure of Aβ40 fibrils extracted from the meninges of human brain tissue from a patient with Alzheimer’s disease (gray; PDB 6SHS). **c**, Comparison of the APP23 (yellow), tg-APP_Swe_ (orange) and ARTE10 (burgundy) Aβ42 fibril fold with human type II Aβ42 filaments (gray; PDB 7Q4M). **d**, Comparison of the murine_Arc_ type I (green) Aβ40 fibril fold with human type I Aβ42 filament fold (gray; PDB 7Q4B).

Interestingly, similarities can be observed between murine type III fibrils and human Arctic (E693G, E22G in Aβ) Aβ filaments^21^ (Fig. 3a). The human Arctic Aβ filament shows two distinct protofilaments (A and B), with each being present twice in the four-protofilament fibril. The main chain trace of murine type III fibrils resembles one protofilament A–B pair of human Arctic Aβ-filaments. The structures, including side chain orientations, are identical between E22/G22 and the C-terminus of Aβ, leading to the same solvent-exposed residues. Moreover, in both cases, the interface is stabilized by salt bridges between D23 and K28. The largest deviation between the two structures can be found in the orientation of the side chains in the N-terminal part up to the single point mutation site (E22G).

### Novel Aβ folds from tg-SwDI mice

For tg-SwDI mice, which harbor the Dutch (E22Q) and Iowa (D23N) mutations within the Aβ sequence, we observed three different polymorphs (Figs. 1 and 2a,e–g). The most dominant polymorph, which we call DI1, accounts for 52% of all reconstructed fibrils and reveals a symmetric dimer (Fig. 2a,e and Supplementary Table 1). The other two polymorphs, labeled DI2 and DI3, consist of a single protofilament and account for 37% and 11%, respectively (Fig. 2a,f,g and Supplementary Table 1).

The 3.3 Å map of DI1 was used to build an atomic model of the ordered core between D1 and V36 (Fig. 2a,e, Extended Data Table 2 and Supplementary Fig. 1d). The two S-shaped protofilaments of DI1 are connected through the extended N-terminus (Fig. 2e). A hydrogen bond between D1 and S26 and a salt bridge between E3 and K28 of the opposing protofilament stabilize the interface between the two subunits. A hydrogen bond between Q15 and N23 stabilizes the first half of the S-turn. This turn is further stabilized by a hydrogen bond between Y10 and a backbone oxygen at N23^i−1^ in the adjacent layer within the same protofilament (denoted by the index i−1).

**Extended Data Fig. 4:**
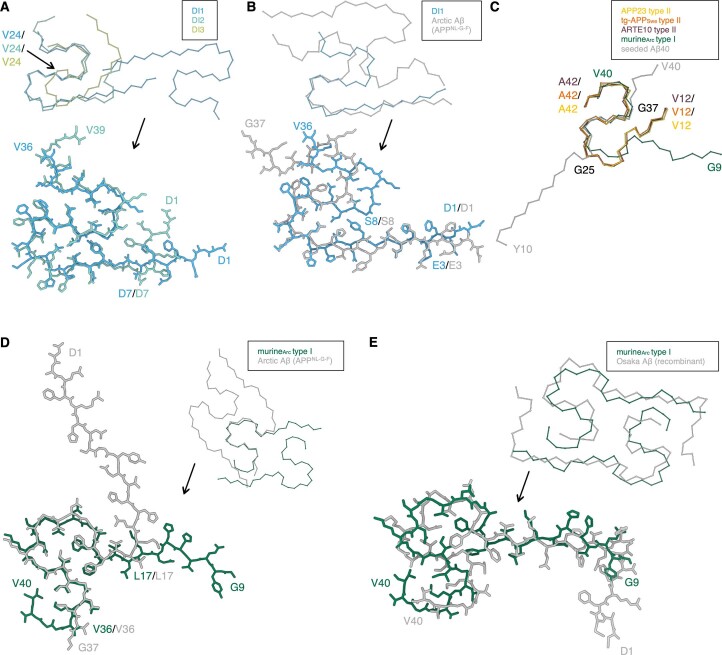
Comparison of murine Aβ fibrils with other structures. (A) Comparison of the main chain trace of DI1 (light blue) with DI2 (teal) and DI3 (light green) (top) and comparison of DI1 and DI2 (bottom). (B) Comparison of DI1 (light blue) and murine Aβ42(E22G) filaments extracted from knock-in APP^NL-G-F^ mice (gray, PDB code: 8BG9). (C) Comparison of APP23 type II (yellow), tg-APP_Swe_ type II (orange), ARTE10 type II (burgundy), tg-APP_ArcSwe_ murine_Arc_ type I (green) fibrils with the cryo-EM structure of Aβ40 fibrils seeded from brain homogenates from cortical tissue of an AD patient (gray, PDB code: 6W0O). (D) Comparison of murine_Arc_ type I Aβ40 (green) with murine Aβ42(E22G) filaments extracted from knock-in APP^NL-G-F^ mice (gray, PDB code: 8BG9). (E) Comparison of murine_Arc_ type I Aβ40 (green) with an NMR structure of recombinant Aβ40 E22Δ fibrils (gray, PDB code: 2MVX).

For DI2 fibrils, an atomic model of residues D1–V39 could be built into the 4.2 Å map (Fig. 2a,f, Extended Data Table 2 and Supplementary Figs. 1e and 3). Except for the N-terminus, the fold is similar to the fold of DI1 fibrils (Fig. 2e,f and Extended Data Fig. 4a). However, although the medium-resolution map allows for straightforward modeling of the main chain, the assignment of the side chains is ambiguous. The presented atomic model represents the best visual fit to the density, and its similarity to the atomic model of DI1 fibrils further adds to its reliability. Furthermore, the model achieves the highest score using a previously presented method for the evaluation of side chain assignments^36^ (Supplementary Fig. 4). In this most probable DI2 model, the N-terminus is fixed in its position by a salt bridge between D1 and K28^i+2^ as well as a hydrogen bond between E3 and S26^i+2^. The second β-sheet found in DI1 is also present in DI2 (Fig. 2a).

For DI3, the atomic model consists of residues A2–G33 (Fig. 2a,g and Supplementary Fig. 5). However, as described for DI2, this atomic model was built into a map with a medium resolution (4 Å) and therefore has limited accuracy. The model is the most likely one based on its fit to the map and the fact that it yielded the highest score in the side chain assignment method (Supplementary Fig. 6). The overall DI3 fold differs from the DI1 fold and aligns only in residues V24–G33 with the DI2 fold (Fig. 2e–g and Extended Data Fig. 4a). Therefore, secondary structure assignments differ from DI1 and DI2, showing two β-sheets in the N-terminal domain (Fig. 2a). The fold of DI3 fibrils is mainly stabilized by a salt bridge between E11 and K28^i+1^ and a hydrogen bond between E11 and S26^i+1^. The C-terminal kink in the structure around K28 is stabilized by a hydrogen bond between N27 and the carbonyl group at G29. Additionally, a hydrophobic cluster around V18, A21 and V24 stabilizes the overall fold (Supplementary Fig. 2f).

Aβ fibrils extracted from the tg-SwDI mouse brain are structurally different from Aβ fibrils extracted from human and APP^NL-F^ mouse brain tissue^18^. Although DI1 fibrils also differ from Aβ40 fibrils extracted from the meninges of patients with Alzheimer’s disease (Fig. 3b)^17^, they share a similar N-terminal fold. Of particular significance is the fact that unlike in most other known Aβ structures, the solvent-exposed N-terminus in human Aβ40 as well as in DI1, DI2 and DI3 Aβ fibrils is ordered. The same similarities can be observed between DI1, DI2 and DI3 Aβ fibrils and Aβ fibrils from APP^NL-G-F^ mice^21,22^ (Extended Data Fig. 4b). The extended N-terminal arm of DI1 overlays with the N-terminus of the human Aβ40 fibril between residues D1 and Y10 and with the murine Arctic Aβ filament between E3 and S8. Moreover, the orientation of these side chains is identical in all structures, suggesting the same degree of solvent-accessibility of the N-terminus.

Finally, in contrast to wild-type Aβ42 fibrils, in which negatively charged and solvent-exposed residues E22 and D23 induce a kink in the main chain, in DI1 and DI2 fibrils, mutant residues Q22 and N23 are interior, in extended conformation (Fig. 2e,f).

### Type II Aβ fibrils from ARTE10, tg-APP_Swe_ and APP23 mice

Aβ fibrils extracted from ARTE10, tg-APP_Swe_ and APP23 mouse brains are composed of Aβ42 and are identical to previously described type II filaments extracted from the brain tissue of patients with Alzheimer’s disease^18^ (Figs. 1, 2a,d,h,i and 3c).

The residues V12–A42 form the ordered core of all three murine type II fibrils (Fig. 2a,d,h,i). Type II fibrils are made of two S-shaped protofilaments, which are related by a C2 symmetry in all three models. Each monomeric subunit is stabilized by two hydrophobic clusters around residues L17, V18, F20, V24, N27, I31 and L34, and residues A30, I32, M35, V40 and A42 (Fig. 2a,d,h,i and Supplementary Fig. 2c,g,h). The interface between the two protofilaments is rather small, involving only two symmetric hydrogen bonds between K28 and A42 of the opposing subunit.

Additionally, as previously discussed^18^, murine models that resemble the type II filament fold overlap in their S-shaped domain partially with seeded Aβ40 fibrils extracted from the cortical tissue of a patient with Alzheimer’s disease^15^ (Extended Data Fig. 4c).

### Murine type I Aβ fibrils from tg-APP_ArcSwe_ mice

Aβ fibrils extracted from tg-APP_ArcSwe_ mouse brain tissue harbor the Arctic mutation (E693G, E22G in Aβ) and have a crossover distance of ~950 Å and a diameter of 90 nm (Fig. 1 and Extended Data Table 1). The fibrils, which we refer to as murine_Arc_ type I Aβ fibrils, consist of two identical S-shaped protofilaments that are related by a pseudo-2_1_ symmetry (Fig. 2j). The 3 Å-resolution map of murine_Arc_ type I Aβ fibrils allowed for atomic model building of the ordered core from G9–V40, in agreement with an observed predominance of Arctic Aβ40 fibrils in the sample (Fig. 2a,j, Extended Data Table 2 and Supplementary Fig. 1i)^26,37^. The S-shaped domain is formed by residues F19–V40, with an associated extended N-terminal arm of G9–V18 that interacts with the C-terminus of the opposing protofilament. The S-shape is stabilized by two hydrophobic clusters around F19, F20, V24 and I31 and around A30, I32, M35 and V40 (Fig. 2j and Supplementary Fig. 2i). The interface between the two protofilaments consists mainly of hydrophobic interactions involving the side chains of Y10, V12, Q15, L17, V36 and V39 at the contact point of the N-terminus of one protofilament and the C-terminus of the opposing protofilament. The fibril center harbors a hydrophobic cavity between the two C-terminal domains of both protofilaments, where two isolated symmetric densities can be observed indicating the presence of additional hydrophobic molecules of unknown identity in the interface.

Murine_Arc_ type I fibrils resemble human type I Aβ42 filaments, which are dominant in extracts from the brain tissues of patients with SAD (Fig. 3d)^18^. In detail, the solvent-accessible surface is almost identical to human type I Aβ42 filaments but the C-terminus is slightly shifted, probably caused by the bound molecules in the hydrophobic fibril cavity. Aβ filaments from APP^NL-G-F^ knock-in mice also carry the Arctic mutation^21,22^, and although APP^NL-G-F^ Aβ filaments and murine_Arc_ type I fibrils share a common substructure between residues L17 and V36, the overall fold and the resulting arrangement of the two protofilaments differs (Extended Data Fig. 4d). Additionally, and in contrast to APP^NL-G-F^ Aβ filaments, in which the mutant residue G22 is hidden in the protofilament interface, the G22 in murine_Arc_ type I Aβ fibrils is exposed to solvents similarly to E22 in human type I filaments. To date, there is no other murine model known that contains predominantly Aβ fibrils that mimic the human type I fold.

The only in vitro preparation that resembles the murine_Arc_ type I fold to a large extent is the nuclear magnetic resonance structure of Aβ40 fibrils with the Osaka mutation (E693Δ, E22Δ in Aβ)^38^ (Extended Data Fig. 4e). In both cases, the mutation (or deletion) of the acidic residue E22 in Aβ40 results in fibrils that are highly similar to human type I filaments. Additionally, it was previously shown that Aβ42 fibrils extracted from the brains of knock-in APP^NL-G-F^ mice, which also harbor the Arctic mutation, do not resemble human type I filaments^21,22^. As discussed for murine type II fibrils, the murine_Arc_ type I fibrils also partially overlap with the cryo-EM structure of brain homogenate seeded Aβ40 (ref. ^15^) (Extended Data Fig. 4c).

### Additional densities in murine Aβ fibrils

**Extended Data Fig. 5:**
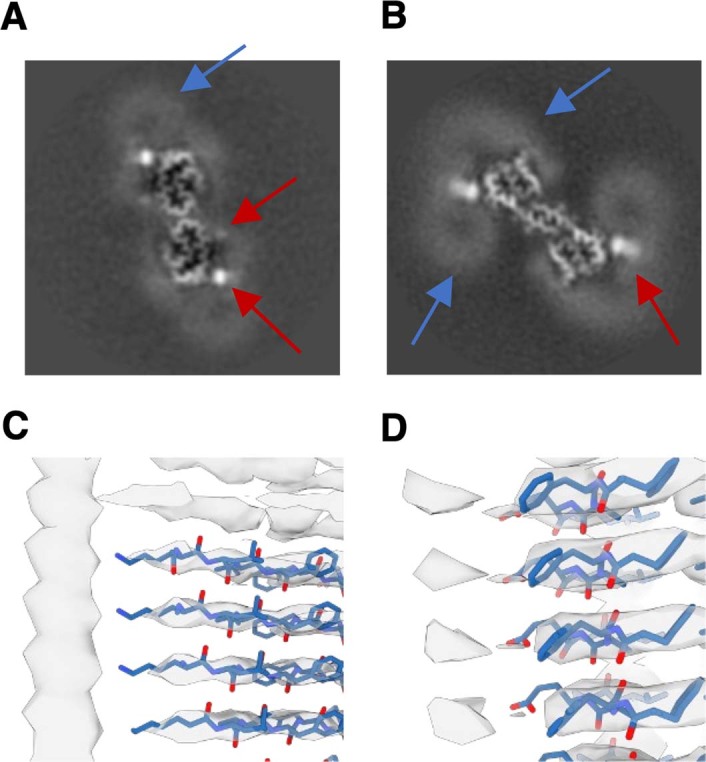
Additional densities bound to murine Aβ fibrils. (A,B) Reconstruction of Aβ fibrils extracted from (A) APP/PS1 and (B) tg-SwDI mice brain. Red arrows indicate localized, strong density, blue arrows indicate micelle-like, weak densities. (C,D) show extra densities close to (C) Lys16 and (D) Phe20/Glu22 in APP/PS1 murine type III Aβ fibrils.

We have observed additional densities on the surface of all murine fibrils. Strong, localized densities can be observed close to K16 in murine type III, DI1, DI2, DI3 and murine type II Aβ fibrils (Figs. 1 and 2 and Extended Data Fig. 5a–c). Moreover, murine type III fibrils show a smaller density close to F20 and E22 (Extended Data Fig. 5d), and in DI3 fibrils, an additional density can be found next to Y10. Similar densities bound to K16 were previously described for APP^NL-G-F^ mice but not for patients with Alzheimer’s disease^18,21^. The observed additional density might be related to bound co-factors or post-translational modifications such as ubiquitination, as was previously described for tau filaments^39^. Weak, micelle-like density of unknown origin bound to the fibril surface is visible in tg-SwDI Aβ fibrils (Fig. 1 and Extended Data Fig. 5b), reminiscent of previously described densities on the surface of alpha-synuclein fibrils^40^.

## Discussion

Here, we extracted Aβ fibrils from brain extracts of six different transgenic mouse models and determined their structure using cryo-EM.

We observed novel Aβ fibril folds in brain extracts from three of the mouse models (APP/PS1, ARTE10 and tg-SwDI). Although murine type III Aβ fibrils extracted from APP/PS1 and ARTE10 mice show some similarities to the human Arctic Aβ filament^21^, their fold has not yet been observed in the brains of patients with Alzheimer’s disease. The tg-SwDI model harbors three mutations (Swedish, Dutch and Iowa)^41,42^, each of which can cause early onset FAD (Swedish) or cerebral amyloid angiopathy (CAA) (Dutch and Iowa)^42^. As DI1, DI2 and DI3 fibril structures differ from the most common Alzheimer’s disease-associated type I and type II filaments^18^, tg-SwDI might not be a suitable model for either SAD or FAD. However, tg-SwDI mice are considered a good model to study CAA^20^ and, indeed, the N-terminus of DI1 fibrils is identical to that of a previously described human Aβ40 polymorph obtained from vascular deposits in the brain meninges associated with CAA^17^. Therefore, our observations support the suitability of the tg-SwDI mouse model for the study of CAA.

Additionally, Aβ fibrils from three of the mouse models (ARTE10, tg-APP_Swe_ and APP23) resemble the human type II filament fold and therefore could, together with the previously described knock-in APP^NL-F^ model^18^, be suitable models for FAD. For example, when the APP23 line was used to assess treatment with the murine parent antibody of aducanumab, a reduction of total plaque area and improvement in spatial memory was seen^43^. However, given our new knowledge of structural differences in Aβ fibrils, the preclinical testing of this treatment might have been more predictive for efficacy in FAD.

Aβ fibrils extracted from tg-APP_ArcSwe_ mice are almost identical to human type I filaments^18^, and therefore here we refer to them as murine_Arc_ type I fibrils. Human type I filaments are mainly found in SAD, which accounts for more than 95% of all patients with Alzheimer’s disease^44^. Compared to human type I filaments, murine_Arc_ type I fibrils show two additional densities of unknown identity in the protofilament interface. The Arctic mutation is also present in knock-in APP^NL-G-F^ mice, but their Aβ(E22G) fibril structure differs from murine_Arc_ type I fibrils and human type I filaments^18,21,22^. In therapeutic research, tg-APP_ArcSwe_ mice were treated with the mAb158 monoclonal antibody, promoting protofibril clearance^45–47^. A humanized version of the mAb158 antibody, named BAN2401 and subsequently renamed lecanemab, showed deceleration of cognitive decline and reduction of amyloid plaque burden in the brains of patients with Alzheimer’s disease^24,48–50^ Accordingly, treatment with an mAb158-based bispecific antibody also showed stronger treatment effects in tg-APP_ArcSwe_ mice compared to knock-in APP^NL-G-F^ mice^51,52^. So far, our murine_Arc_ type I structure is the only murine fibril structure that resembles the human type I filament fold and, therefore, tg-APP_ArcSwe_ might be a suitable model to predict which drug candidate will show efficacy in SAD. Clinical success of a therapeutic depends on multiple factors, but the fact that lecanemab is efficacious in both preclinical evaluation in tg-APP_ArcSwe_ mice and in clinical evaluation might at least in part be explained by the structural similarity between murine_Arc_ type I fibrils and human type I filaments mainly found in brains of patients with SAD^24^. Moreover, recent investigations indicating that lecanemab binds not only to intermediately sized soluble aggregates but also to ‘diffusible Aβ fibrils’, whose structure is identical to that of Aβ fibrils found in insoluble plaques^26,27,48^, provide a further molecular explanation to the success of this therapeutic anti-Aβ antibody.

The [^11^C]Pittsburgh compound B (PiB) and the later-developed fluorine-18 (^18^F) radiolabeled analogs are commonly used PET tracers to detect Alzheimer’s disease pathology in the living brain. A positive amyloid PET scan has served as an inclusion criterion in anti-Aβ immunotherapy trials, and a reduction in PET signal intensity has been interpreted as successful removal of brain amyloid plaque and, thus, included as a secondary endpoint in the clinical trials. PET imaging performed in the tg-APP_ArcSwe_ mouse model with [^11^C]PiB visualizes amyloid pathology^26^. This observation is also in line with our observation that the murine_Arc_ type I structure found in the tg-APP_ArcSwe_ model resembles the human type I filament fold that is mainly found in the brains of patients with SAD. It is believed that the ability of [^11^C]PiB to detect pathology depends on differences in the structure of amyloid plaques and Aβ fibrils therein^53–56^. For example, it has been shown that the tg-APP_ArcSwe_ model exhibits higher [^11^C]PiB binding than the APP^NL-G-F^ model^57^, whose purified Aβ structures differ from human type I and type II filaments. Furthermore, [^11^C]PiB also works effectively in the ARTE10, tg-APP_Swe_ and APP23 mouse models, which all show an Aβ fibril fold similar to human type II filaments as long as the mice exhibit high total brain Aβ levels. Interestingly, a recent study that used the ^18^F-labeled amyloid PET tracer florbetaben to directly compare the APP/PS1 and the ARTE10 mouse models showed that the ARTE10 mice, which mainly contain type II fibrils, are more suitable for amyloid PET owing to their dense-cored plaques and overall higher plaque load compared to the APP/PS1 mouse model^56^. Yet [^11^C]PiB does not work effectively in every mouse model. For example, the APP/PS1 model does not display any positive amyloid PET signals, which may be explained by the fact that such mice contain the murine type III fibrils that are similar to those found in patients with the Arctic mutation, who are ‘PET-negative’^21,35^ in spite of having massive Aβ-deposition post mortem^58,59^.

Therapeutic approaches that succeeded in animals and failed to produce positive outcomes in humans^20,29–34^ may have overlooked the possibility that animal models might not contain the relevant molecular drug targets for SAD; that is, that the associated Aβ fibrils might not present the same folds and surfaces. Considering that most patients with Alzheimer’s disease have a sporadic background, one can speculate that this might be one important reason why the failure rate of clinical trials has been so high^28^. Structural studies of Aβ fibrils from animal models and their comparison to human Aβ fibrils provide a more detailed understanding of the nature of the molecular targets and may thereby help us to identify the most adequate animal model for the development of novel Alzheimer’s disease treatments and PET tracers targeting amyloid deposits.

## Methods

### Animals

In the present study, the following mouse lines were used for experimentation including immunohistochemistry, negative stain sample screening, immunogold negative stain and cryo-EM:

APP/PS1 (APPswe/PSEN1dE) (heterozygous; *n* = 4 (3 males, 1 female); 27–33 months old) on a C57BL/6;C3H background (strain name B6.Cg-Tg(APPswe,PSEN1dE9)85Dbo/Mmjax) are well described in terms of their behavioral and pathological characteristics^61–63^. Depending on the used protocol, APP/PS1 mice develop (contextual and spatial) cognitive deficits by 7 months of age. Aβ plaques can be detected by 6 months of age in the hippocampus and cortex, followed by a pronounced gliosis. Abundant Aβ plaques and gliosis are prominent at 12 months of age. Four heterozygous APP/PS1 mice brains were used in this study.

ARTE10 (homozygous; *n* = 1 (female); 24 months old) mouse on a C57Bl/6 background (strain name B6.CBA-Tg(Thy1-PSEN1*M146V,-APP*Swe)10Arte) was a generous gift from Taconic Biosciences. The mice express APPswe (APP KM670/671NL) and PS1-M146V under Thy1.1 regulatory sequences, which leads to the development of a progressive plaque pathology and CAA starting around the age of 3 months^64^.

Tg-SwDI mice (heterozygous; *n* = 4 (all male); 26–29 months old) on a C57BL/6 background (strain name C57BL/6-Tg(Thy1-APPSwDutIowa)BWevn/Mmjax) were first introduced in 2004 as a model to study CAA in Alzheimer’s disease^42,65^. Cognitive deficits and Aβ plaques with associated gliosis can be detected by 3 months of age, increasing and manifesting with age.

APP23 mice (heterozygous; *n* = 2 (all male); 21 months old) are on a C56BL/6 background (strain name B6.Cg-Tg(Thy1-APP)3Somm/J) and have a sevenfold overexpression of mutant human APP_751_ bearing the pathogenic Swedish mutation. Aβ deposit starts at 6 months of age and increases in size and number with age^66^. APP23 mice also develop CAA^67^.

Tg-APP_ArcSwe_ (heterozygous; *n* = 1 (male); 18 months old) and tg-APP_Swe_ (heterozygous; *n* = 2 (all male); 22 months old) are maintained on a C57BL/6 background^68^. Tg-APP_ArcSwe_ mice harbor the Swedish and the Arctic APP mutations and develop plaque pathology starting at around 6 months of age^68,69^, while Tg-APP_Swe_ mice that harbor the Swedish mutation have a later onset of plaque pathology starting at 10–12 months of age, increasing with rapidly with age.

APP/PS1, ARTE10, tg-SwDI and APP23 experiments were performed in accordance with the German Law on the protection of animals (TierSchG §§7–9). Breeding of APP/PS1 mice was approved by a local ethics committee (Landesamt für Natur, Umwelt und Verbraucherschutz Nordrhein-Westfalen (LANUV), Az: 84-02.04.2014.362) before the start of the study. APP/PS1 and tg-SwDI mouse lines were purchased from the Jackson Lab (JAX MMRRC Stock no. 034829 or JAX MMRRC Stock no. 034843). The tg-APP_ArcSwe_ and tg-APP_Swe_ mice were bred under the ethical permit 5.8.18-20401/20, approved by the Uppsala County Animal Ethics Board. All mice were kept and bred under controlled conditions with a 12:12 h light:dark cycle, 54% humidity, a temperature of 22 °C as well as food and water ad libitum.

### Brain tissue characterization

Brain tissue from the tg-APP_Swe_, tg-APP_ArcSwe_ and the ARTE10 mouse models has been extensively characterized in previous studies^37,56,64,68,70,71^.

The remaining APP/PS1, tg-SwDI and APP23 mouse lines were immunohistochemically stained as follows. In brief, after cervical dislocation, the brains were snap-frozen in isopentane and cut into 20 µm sagittal sections with a microtome. The sections were fixed with 4% paraformaldehyde (PFA) in TRIS-buffered saline (TBS) for 10 min at 21 °C. The sections were then washed three times with 1% Triton in TBS (TBST) for 5 min and further incubated in 70% formic acid for 5 min at room temperature for antigen retrieval. The sections were again washed with TBST before incubation with primary antibody overnight at 4 °C in a humidified chamber (6E10 (BioLegend, Alexa Fluor 594 anti-β-Amyloid, 803018, lot no. B309351) and 4G8 (BioLegend, 800703, lot no. B239200), both diluted 1:500 in TBST with 1% BSA). The next day, the tissue sections were washed with TBST before incubation with the secondary antibody (only 4G8, goat anti-mouse antibody, Alexa Fluor 488, Invitrogen, diluted 1:300 in TBST and 1% BSA) for 1 h at room temperature. For cell nuclei staining, the sections were washed again with TBST before incubation with DAPI for 5 min. Subsequently, the sections were washed three times with TBST before mounting (Fluoromount Aqueous Mounting Medium, Sigma-Aldrich). Images were taken with a LMD6000 microscope (Leica Camera) with a DFC310 FX camera (Leica Camera).

### Extraction of Aβ fibrils

Aβ fibril extraction was essentially based on a published procedure^18^. In brief, non-fixed mouse brain tissue was snap-frozen in −80 °C cold isopentane and stored at −80 °C before experimentation. Between 0.4 and 0.6 g of brain tissue was thawed and manually homogenized in 20× volume (w/v) of extraction buffer (10 mM Tris-HCl, pH 7.5, 0.8 M NaCl, 10% sucrose, 1 mM EGTA) by applying 300 strokes using a Dounce glass tissue grinder. Subsequently, 10% sarkosyl diluted in _d_H_2_O (Sigma-Aldrich) was added to the homogenate to a final sarkosyl concentration of 2% and was thoroughly mixed 30 times by pipetting up and down. After 1 h incubation at 37 °C, the homogenate was centrifuged at 10,000×*g* for 10 min at 4 °C and the resulting supernatant was further ultracentrifuged at 100,000×*g* for 60 min at 4 °C (Beckman Coulter Optima MAX-XP, TLA55 fixed-angle rotor). After removal of the supernatant, extraction buffer (1 ml g^–1^ original tissue mass) was added to the pellet and mixed, followed by 5,000×*g* centrifugation for 5 min at 4 °C. The supernatant was then diluted threefold in dilution buffer (50 mM Tris-HCl, pH 7.5, 0.15 M NaCl, 10% sucrose, 0.2% sarkosyl) and ultracentrifuged at 100,000×*g* for 30 min at 4 °C. The resulting supernatant was discarded and resuspension buffer (20 mM Tris-HCl, pH 7.4, 50 mM NaCl) was added (100 µl g^–1^ original tissue mass) to the sarkosyl insoluble Aβ fibril-rich pellet. The pellet was used for further negative staining, immunogold labeling and cryo-EM analysis.

We noticed that the fibril extraction protocol was sensitive to changes in temperature, sarkosyl concentration and frequency of homogenization; therefore, the procedure was optimized accordingly.

### Negative stain electron microscopy

A total of 2 µl of the final sarkosyl insoluble fraction, consisting of a homogeneous mixture of the final pellet after fibril extraction and resuspension buffer, was applied onto a glow-discharged 300 mesh carbon-coated copper grid (EM Sciences, ECF300-Cu). The sample was incubated for 2 min and carefully blotted off with filter paper. The sample was then washed once with _d_H2O and blotted off immediately. A total of 2 µl of 1% (w/v) uranyl acetate (UrAc) was applied on the top of the grid, following a 1 min incubation. The UrAc was removed with filter paper and the grid was air-dried. Transmission electron microscopy images were acquired using a ThermoFisher Scientific Talos 120C at an acceleration voltage of 120 kV. Images were collected on a 4k × 4k Ceta 16 M CEMOS camera using Thermo Scientific Velox Software.

### Immunogold negative stain electron microscopy

Immunogold negative-stain grids for electron microscopy were prepared as previously described^72^. In brief, 3 µl of the final pellet containing the extracted Aβ fibrils were placed on a glow-discharged 300 mesh carbon-coated copper grid (EM Sciences, ECF300-CU) for 2 min. The sample was washed once with _d_H20 and placed in blocking buffer for 15 min, following incubation with Nab228 (Sigma-Aldrich, A8354, lot no. 0000121536) primary antibody diluted in blocking buffer (diluted 1:1000; final concentration of 2 µg ml^–1^) for 1–2 h. Then, the grid was washed with washing buffer and was incubated with 6 nm gold-conjugated anti-mouse secondary antibody (diluted 1:20 in blocking buffer, Abcam) for 1 h. The grid was washed five times with washing buffer and three times with _d_H20 before staining with 1% (w/v) uranyl acetate for 1 min. The sample was air-dried, and electron microscopy images were acquired as described above. Immunogold negative stain for electron microscopy confirmed that the purified fibrils were indeed Aβ fibrils (Extended Data Fig. 2).

### Cryo-EM image acquisition and data preprocessing

For cryo-EM imaging, 2–3 µl of Aβ fibril sample from a single mouse brain was applied to holey carbon grids (Quantifoil 1.2/1.3, 300 mesh), blotted with filter paper for 3–5 s and plunge-frozen in liquid ethane using a ThermoFisher Scientific Vitrobot Mark IV, set at 95% humidity and 4 °C temperature. Data acquisition was performed on a ThermoFisher Scientific Talos Arctica microscope operating at 200 kV using a Gatan BioQuantum K3 detector in counting mode with a Gatan BioQuantum energy filter with a slid width of 20 eV, and on a ThermoFisher Scientific Titan Krios G4 operating at 300 kV using a Falcon 4 detector in counting mode. The automated collection was directed by EPU data collection software. Further details are given in Extended Data Table 2.

For helical reconstruction of all datasets, gain-corrected movie frames were aligned and summed into single micrographs on-the-fly using Warp v. 110Beta^73^. CTF estimation was performed using CTFFIND4.1 (ref. ^74^).

### Helical reconstruction

Helical reconstruction was performed using the helical reconstruction methods in RELION 3.1.0 (refs. ^75,76^). The helical image processing follows the procedures previously described^77^. For all datasets, fibrils were picked automatically using crYOLO 1.8.4 (refs. ^78,79^). Automatically picked filaments were extracted at a larger box size of 754 pix, 772 pix or 800 pix downscaled to 200 pix. Reference-free 2D classification was performed to separate different polymorphs and to discard low-quality particle images.

For ARTE10 murine type II, ARTE10 murine type III, DI2, DI3, tg-APP_Swe_ murine type II, APP23 murine type II and murine_Arc_ type I, a featureless cylinder was used as the initial 3D reference. For APP/PS1 murine type III and DI1, an initial 3D reference was computed de novo from multiple 2D class averages assuming a helical rise of 4.75 Å and a twist value calculated from the crossover distance of each fibril observed from larger box 2D class averages, using *relion_helix_inimodel2d*^77^. Cylinders were initially low-pass filtered to 40 Å, and reconstructed de novo initial models were low-pass filtered to 8–10 Å depending on their quality. Iterative 2D and 3D classification was used to obtain a homogeneous high-quality subset of particles for each fibril polymorph. Helical parameters were refined iteratively during 3D refinement in between classification steps. 3D auto-refinement and subsequent post-processing was performed to compute the final maps and to calculate the resolution according to gold-standard Fourier shell correlations at 0.143, applying a soft-edged solvent mask. For APP/PS1 murine type III, ARTE10 murine type III, DI1, DI2 and tg-APP_Swe_ type II fibrils, VISDEM sharpening^80^ was used instead of automatic B-factor sharpening. Additional image processing information can be found in Extended Data Table 2.

While refining the tg-APP_ArcSwe_ fibril, we initially obtained low-quality 3D density maps. A plausible explanation was that some parts of the fibril may be unstructured or that some molecules are attached to the fibril, which, in turn, hampered image alignment during 3D refinement. Interestingly, closer inspection of the 2D class averages also revealed fuzzy edges at the fibril periphery (Extended Data Fig. 3f), supporting our hypothesis. To overcome this limitation, we followed the masked refinement with the signal subtraction procedure^81^ during 3D refinement. To do so, we initially refined the fibril until a coarse filament fold became visible in the map’s cross-section. We then manually edited the density map using the eraser tool in UCSF Chimera 1.15 (ref. ^82^) to keep only the fibrillar part of the map. This manually edited map was then used to create a narrow mask (extended by two pixels and further extended by a soft edge of three pixels), extending 90% of the *Z*-length. This mask was used for the signal subtraction, keeping only the fibrillar part in the particle images, and for the following masked 3D refinement runs. Initially, we performed a masked 3D refinement run with three classes. Then, selecting only the highest populated class, we continued a masked 3D refinement with a single class. The helical parameters were optimized after the amyloid-characteristic stacked β-strands became visible. Finally, we performed a masked 3D auto-refinement, followed by standard RELION post-processing to compute the final maps and to calculate the resolution according to gold-standard Fourier shell correlations at 0.143.

Statistics on the distribution of different polymorphs are given in Supplementary Table 1. The term “unassigned’ refers to particles that were originally picked by crYOLO but that were either false positives (carbon, beam edge and so forth) or that were too noisy, heterogenous and could not be used for further structure determination.

### Model building and refinement

For APP/PS1 murine type III fibrils and all tg-SwDI polymorphs, atomic models were built de novo into the computed cryo-EM reconstructions using COOT 0.8.9.2 (ref. ^83^). Side chain rotamers were refined manually monitoring Ramachandran outliers and clash scores using MolProbity 4.5.1 (ref. ^84^). All models were refined using an iterative procedure of refinement in PHENIX 1.20.1 (ref. ^85^) and manual modeling in COOT and ISOLDE 1.3 (ref. ^86^). For the medium-resolution tg-SwDI DI2 and DI3 reconstructions, the correctness of the de novo built atomic models sequence assignment was additionally verified following a previously presented method^36^. For DI2, 39 residues were visible in the density map. Accordingly, two polyalanine backbones, each containing 39 residues, were manually built into the density map in forward and backward directions using COOT. The eight possible sequences were assigned to the backbone using SCWRL 4.0 (ref. ^87^). The resulting eight atomic models were energy-minimized with CNS 1.3 (ref. ^88^) and refined into the density map using DireX 0.7.1 (ref. ^89^). The DireX refinement was performed using a density map low-pass filtered to 4.2 Å. The atomic models were ranked based on their *C*_free_ value^90^, which describes the cross-validated real-space map cross-correlation coefficient computed from the density map filtered with a bandpass of 3.2–4.2 Å resolution (Supplementary Fig. 4); this range was not used for the structure refinement. By contrast, the *C*_work_ value is the map cross-correlation from the low-pass filtered maps, which is the information that was used during refinement. For DI2, the highest *C*_free_ value is reached for the D1–V39 model. For DI3, 32 residues were visible in the density map. Analogous to DI2, two polyalanine backbones, each containing 32 residues, were manually built into the density map in forward and backward directions using COOT. The 22 possible sequences were assigned to the backbone using SCWRL. The resulting 22 atomic models were energy-minimized with CNS and refined into the density map using DireX using a resolution cut-off at 4.0 Å. The *C*_free_ value computed from the density maps filtered with a bandpass of 3.0–4.0 Å was used to rank the atomic models (Supplementary Fig. 6). For DI3, the highest *C*_free_ value is reached for the A2–G33 model. For both DI2 and DI3, the most probable atomic models that achieved the highest *C*_free_ value (as well as the highest *C*_work_ value) in the independent DireX analysis show a sequence assignment identical to the corresponding de novo built atomic models.

For ARTE10 murine type III fibrils, the atomic model of APP/PS1 murine type III filaments was fitted into the density and refined using COOT, ISOLDE and PHENIX. For ARTE10, tg-APP_Swe_ and APP23 murine type II fibrils, an atomic model of previously determined human type II Aβ filaments (ref. ^18^; PDB 7Q4M) was fitted into the density maps and refined using COOT and PHENIX. For tg-APP_ArcSwe_ fibrils, an atomic model of previously determined human type I Aβ filaments (ref. ^18^; PDB 7Q4B) was fitted into the density maps and refined using COOT and PHENIX. In all cases, five layers of the fibril model were built and NCS restraints between all chains were used during the refinements in PHENIX. ChimeraX^91^ was used for molecular graphics and analyses. Additional information on the final models can be found in Extended Data Table 2.

### Statistics and reproducibility

Sample sizes (*n*) are indicated in the Extended Data figure legends and in the Extended Data Tables. No statistical methods were used to pre-determine sample sizes, but our sample sizes are similar to those reported in previous publications (refs. ^18,21,22,27^). Pre-established common image classification procedures (ref. ^92^) were used to select the particle images containing the highest resolution information for high-resolution cryo-EM reconstruction. Details on the number of selected images are given in Extended Data Table 2. Data distribution was assumed to be normal but this was not formally tested. Data collection and analysis were not randomized and were not performed blind to the conditions of the experiments.

## Data and materials availability

Cryo-EM maps have been deposited to the Electron Microscopy Data Bank (EMDB) and the Protein Data Bank (PDB) under the following accession numbers: EMD-16944 (PDB 8OL3) for murine type III Aβ42 fibrils from APP/PS1, EMD-16960 (PDB 8OLO) for murine type III Aβ fibrils from ARTE10, EMD-16949 (PDB 8OL5) for type II Aβ42 fibrils from ARTE10, EMD-16959 (PDB 8OLN) for DI1 Aβ fibrils from tg-SwDI, EMD-16957 (PDB 8OLG) for DI2 Aβ fibrils from tg-SwDI, EMD-16961 (PDB 8OLQ) for DI3 Aβ fibrils from tg-SwDI, EMD-16952 (PDB 8OL6) for type II Aβ42 fibrils from tg-APP_Swe_, EMD-16942 (PDB 8OL2) for type II Aβ42 fibrils from APP23 and EMD-16953 (PDB 8OL7) for murine_Arc_ type I Aβ40 fibrils from tg-APP_ArcSwe_. Raw cryo-EM multi-frame micrographs were deposited to the Electron Microscopy Public Image Archive (EMPIAR) for Aβ fibrils purified from tg-SwDI mouse brain tissue under accession code EMPIAR-11680.

### Reporting summary

Further information on research design is available in the Nature Portfolio Reporting Summary linked to this article.

## Online content

Any methods, additional references, Nature Portfolio reporting summaries, source data, extended data, supplementary information, acknowledgements, peer review information; details of author contributions and competing interests; and statements of data and code availability are available at 10.1038/s41593-023-01484-4.

### Supplementary information

Supplementary Information Supplementary Figs. 1–6, Supplementary Table 1.

Reporting Summary

## Figures and Tables

**Extended Data Table 1:**
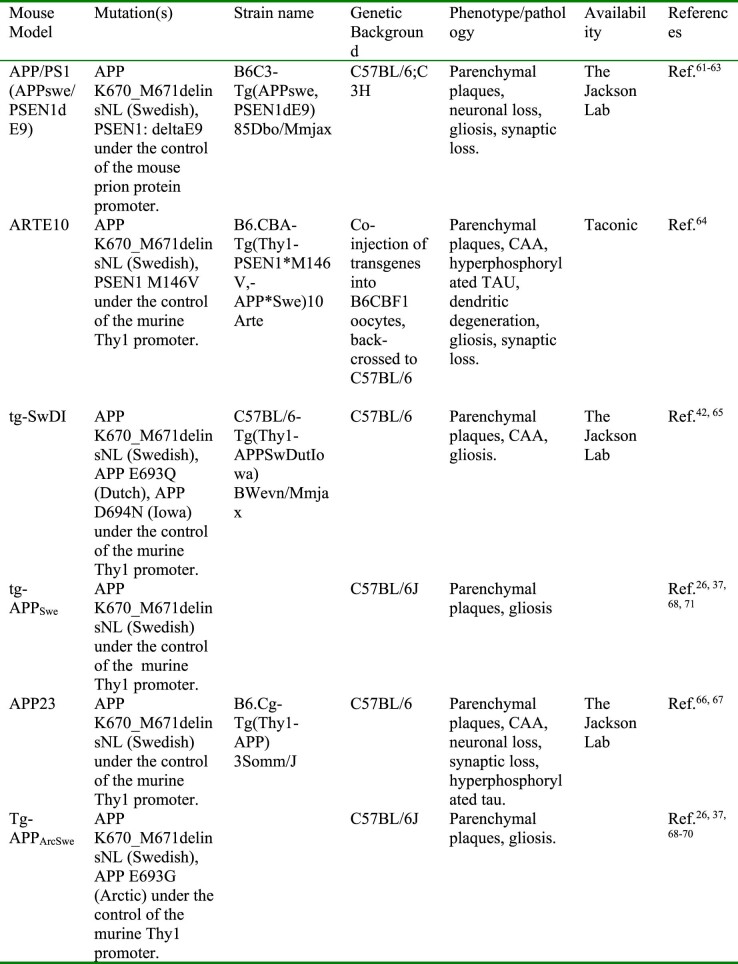
Summary of mouse models

**Extended Data Table 2:**
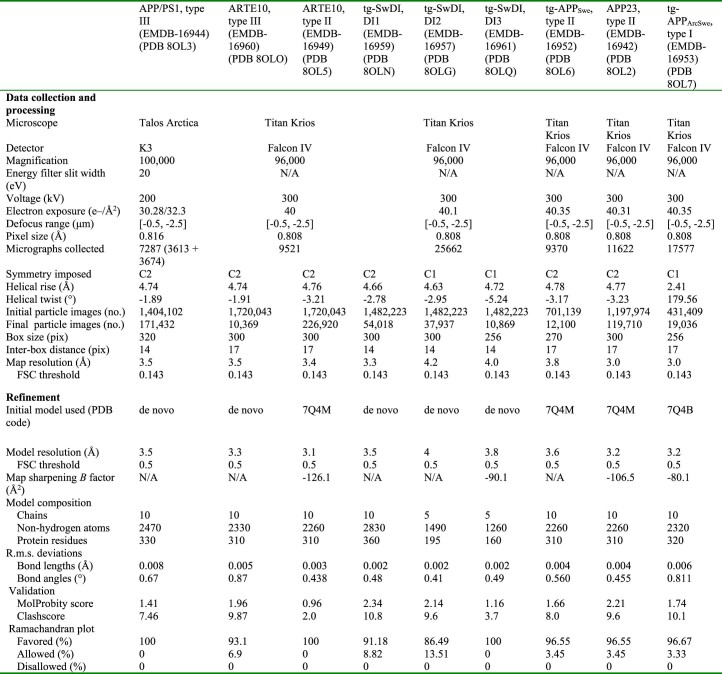
Cryo-EM data collection, refinement and validation statistics
