# Supplementary material for: Cryo-EM of Aβ fibrils from mouse models find tg-APPArcSwe fibrils resemble those found in patients with sporadic Alzheimer’s disease
Source: Nat Neurosci. 2023 Nov 16;26(12):2073–80. doi: 10.1038/s41593-023-01484-4 (PMC10689242; doi:10.1038/s41593-023-01484-4)
Supplement: Supplementary file 1 — Supplementary Figs. 1–6, Supplementary Table 1. [file 41593_2023_1484_MOESM1_ESM.pdf]

nature neuroscience

Article https://doi.org/10.1038/s41593-023-01484-4

Cryo-EM of Aβ fibrils from mouse models find tg-APP$_{ArcSwe}$ fibrils resemble those found in patients with sporadic Alzheimer's disease

In the format provided by the authors and unedited

Cryo-EM of Aβ Fibrils from Mouse Models find tg-APP$_{ArcSwe}$ fibrils resemble those found in sporadic Alzheimer's disease patients

SUPPLEMENTARY DATA

Mara Zielinski[1,2*], Fernanda S. Peralta Reyes[3*], Lothar Gremer[1,2,3§], Sarah Schemmert[1], Benedikt Frieg[1,2], Luisa U. Schäfer[1,2], Antje Willuweit[4], Lili Donner[5], Margitta Elvers[5], Lars N. G. Nilsson[6], Stina Syvänen[7], Dag Sehlin[7], Martin Ingelsson[7,8,9], Dieter Willbold[1,2,3§], Gunnar F. Schröder[1,2,10§]

[1]Institute of Biological Information Processing, Structural Biochemistry (IBI-7), Forschungszentrum Jülich, Jülich, Germany

[2]JuStruct, Jülich Center for Structural Biology, Forschungszentrum Jülich, Jülich, Germany

[3]Institut für Physikalische Biologie, Heinrich Heine University Düsseldorf, Düsseldorf, Germany

[4]Institute of Neuroscience and Medicine, Medical Imaging Physics (INM-4), Forschungszentrum Jülich GmbH, Jülich, Germany

[5]Department of Vascular- and Endovascular Surgery, University Hospital Düsseldorf, Heinrich-Heine University, Düsseldorf, Germany

[6]Department of Pharmacology, Institute of Clinical Medicine, University of Oslo and Oslo University Hospital, Oslo, Norway

[7]Department of Public Health and Caring Sciences, Molecular Geriatrics, Rudbeck Laboratory, Uppsala University, Uppsala, Sweden

[8]Krembil Brain Institute, University Health Network, Toronto, Ontario, Canada

[9]Tanz Centre for Research in Neurodegenerative Diseases, Departments of Medicine and Laboratory Medicine & Pathobiology, University of Toronto, Toronto, Ontario, Canada

[10]Physics Department, Heinrich Heine University Düsseldorf, Düsseldorf, Germany.

[§]corresponding authors.

[*]These authors contributed equally to this work

LUS current address: Life and Medical Sciences (LIMES) Institute, University of Bonn, Germany

This file includes:

Supplementary Figures 1-6

Supplementary Table 1

Supplementary figures

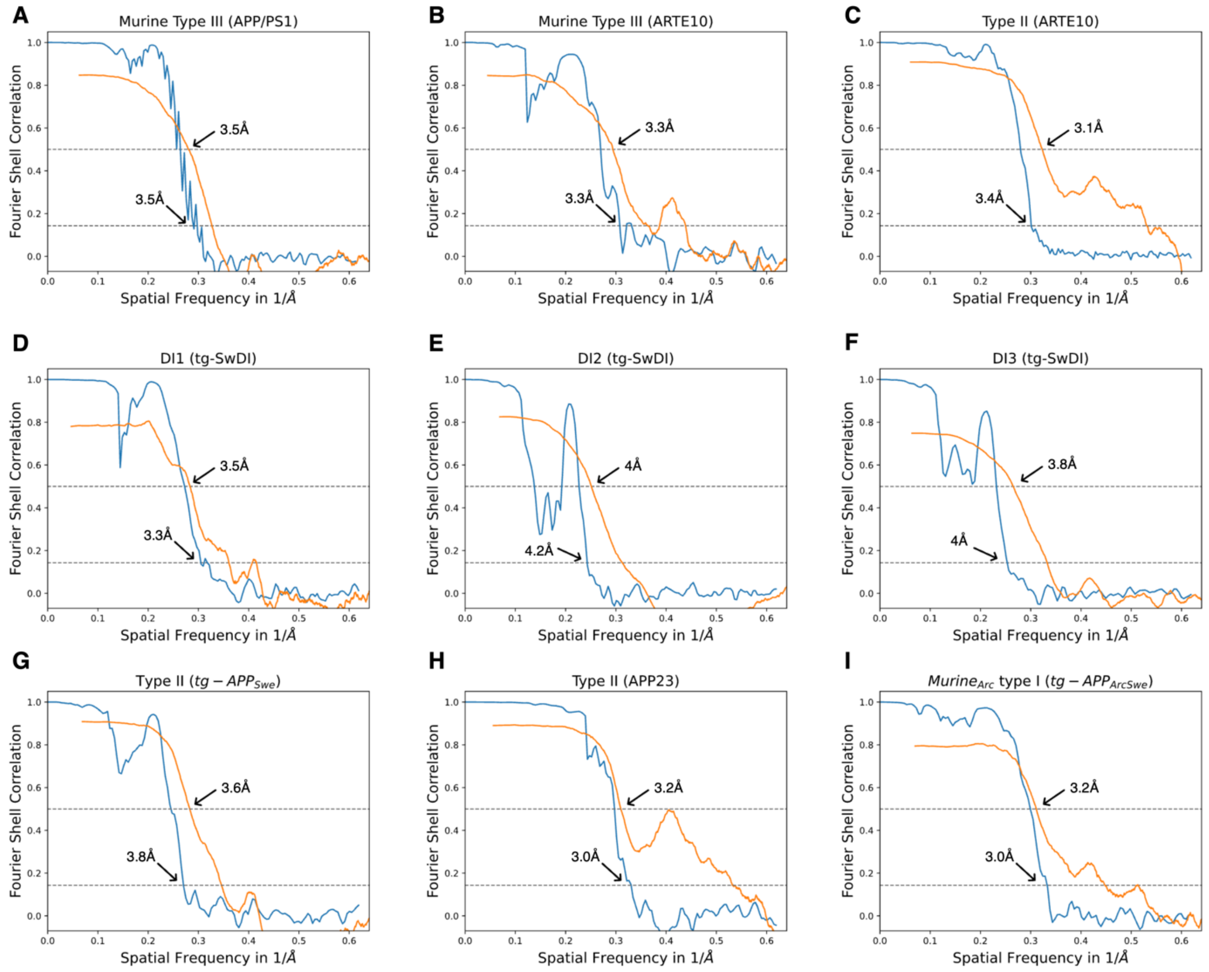

Figure S1: FSC curves for cryo-EM maps and structures of (A) APP/PS1 murine type III, (B) ARTE10 murine type III, (C) ARTE 10 type II, (D) DI1, (E) DI2, (F) DI3, (G) tg-APP$_{Swe}$ type II, (H) APP23 type II, and (I) tg-APP$_{ArcSwe}$ murine$_{Arc}$ type I fibrils. FSC curves for two independently refined half maps are shown in blue; FSC curves for the refined atomic model against the final cryo-EM map in orange.

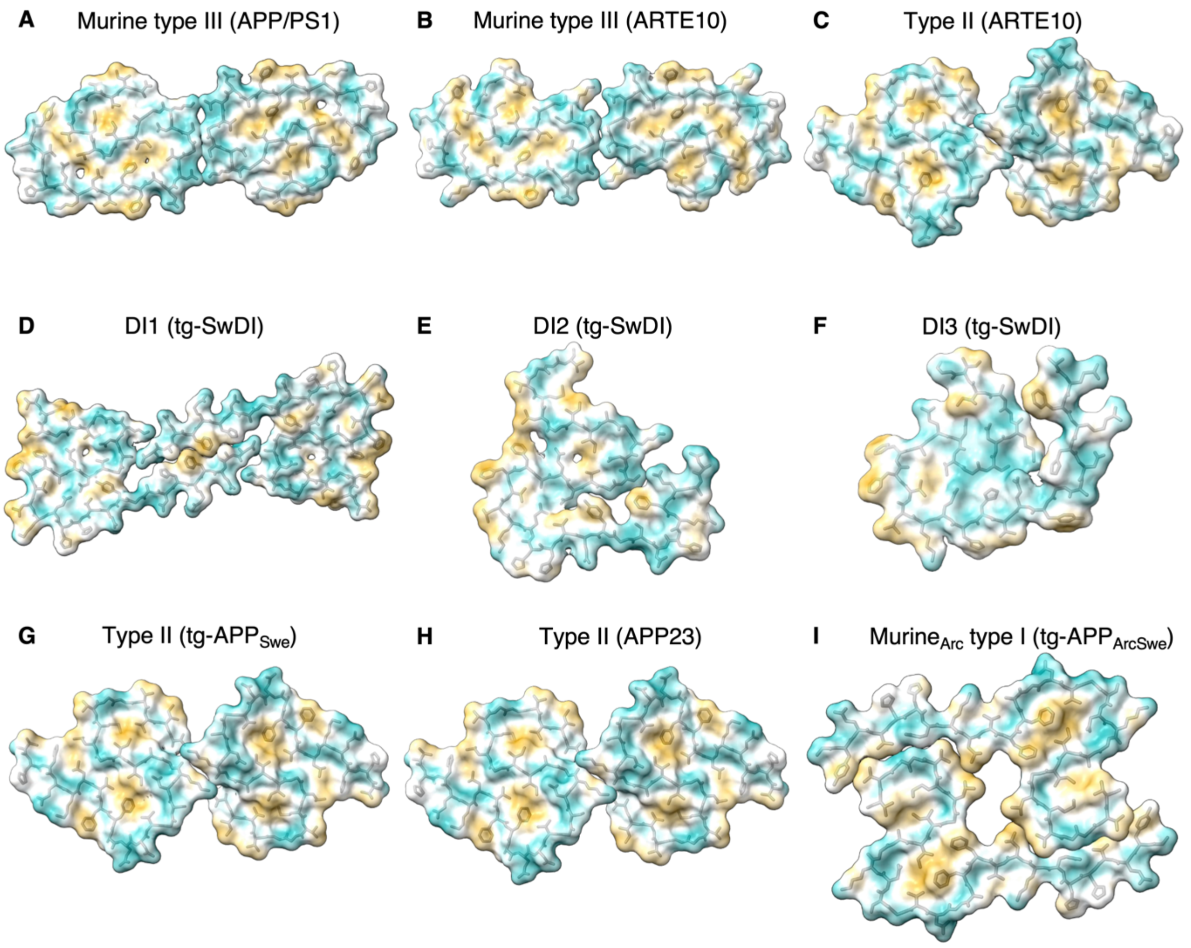

Figure S2: Molecular lipophilicity potential (MLP) maps for (A) APP/PS1 murine type III, (B) ARTE10 murine type III, (C) ARTE10 type II, (D) DI1, (E) DI2, (F) DI3, (G) tg-APP$_{Swe}$ type III, (H) APP23 type II, and (J) murine$_{Arc}$ type I. Coloring on the surface ranging from cyan (most hydrophilic) to white to yellow (most lipophilic/hydrophobic).

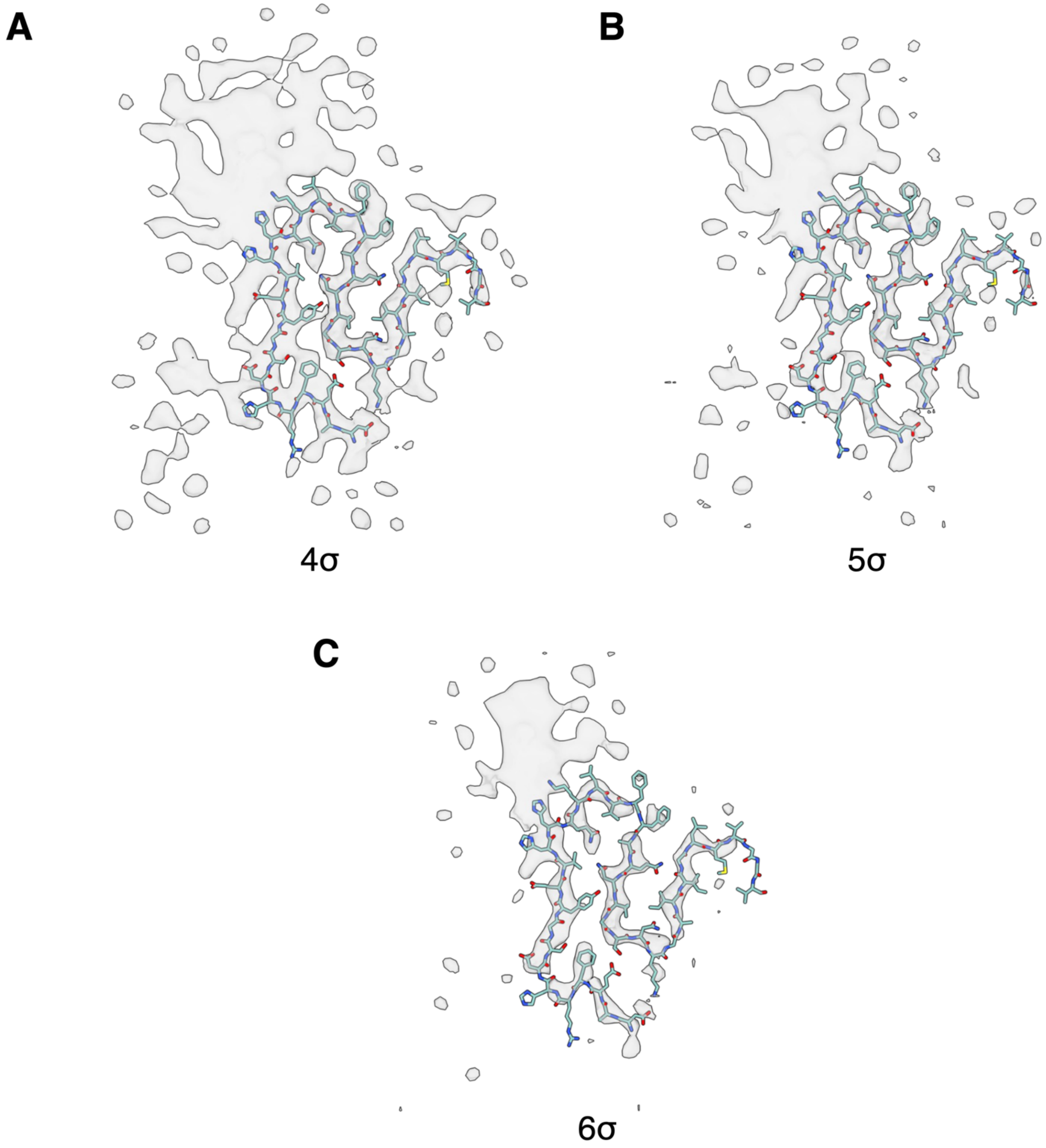

Figure S3: DI2 density map at different thresholds. At (A) 4σ, (B) 5σ, and (C) 6σ

A

	Cfree	Cwork	Sequence assignment
1	0.10151	0.84414	D A E F R H D S G Y E V H H Q K L V F F A E D V G S N K G A I I G L M V G G V V I A
2	0.09942	0.78261	A I V V G G V M L G I I A G K N S G V D E A F F V L K Q H H V E Y G S D H R F E A D
3	0.07265	0.80838	D A E F R H D S G Y E V H H Q K L V F F A E D V G S N K G A I I G L M V G G V V I A
4	0.07157	0.80478	D A E F R H D S G Y E V H H Q K L V F F A E D V G S N K G A I I G L M V G G V V I A
5	0.07024	0.82736	D A E F R H D S G Y E V H H Q K L V F F A E D V G S N K G A I I G L M V G G V V I A
6	0.06892	0.78052	A I V V G G V M L G I I A G K N S G V D E A F F V L K Q H H V E Y G S D H R F E A D
7	0.06431	0.79885	A I V V G G V M L G I I A G K N S G V D E A F F V L K Q H H V E Y G S D H R F E A D
8	0.06263	0.79658	A I V V G G V M L G I I A G K N S G V D E A F F V L K Q H H V E Y G S D H R F E A D

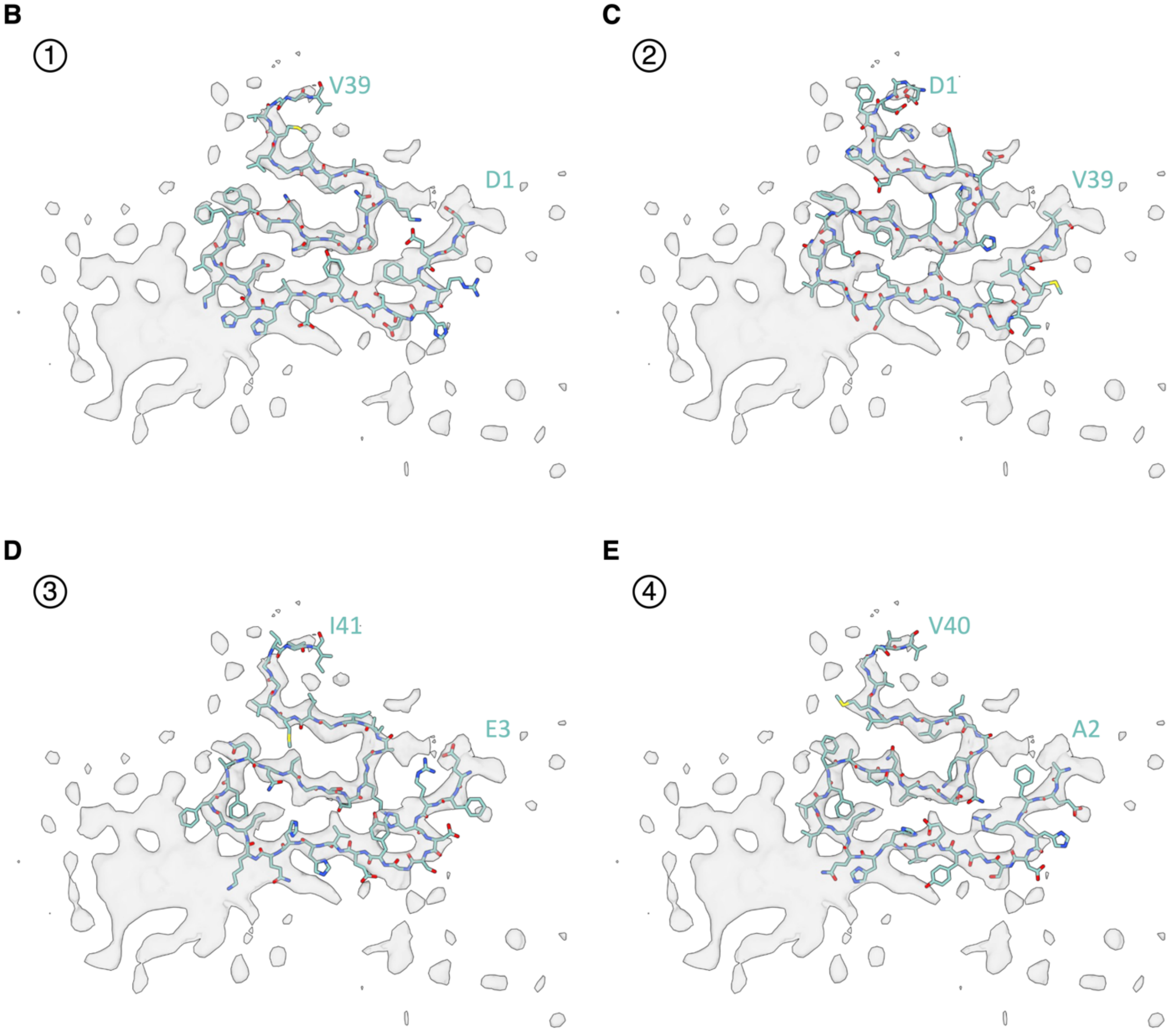

Figure S4: DireX analysis of DI2. (A) The table contains the C_{work} and C_{free} values from the DireX fitting of all possible eight sequence assignments (black box) in both possible C_{α}-chain directions into the DI2 density maps**.** The C_{free} value was used to rank the results. The N-terminus of the sequence is coloured in grey, the C-terminus is coloured in green. (B) The most favourable sequence fit. (C)-(E) The 2nd to 4th most favourable sequence fit.

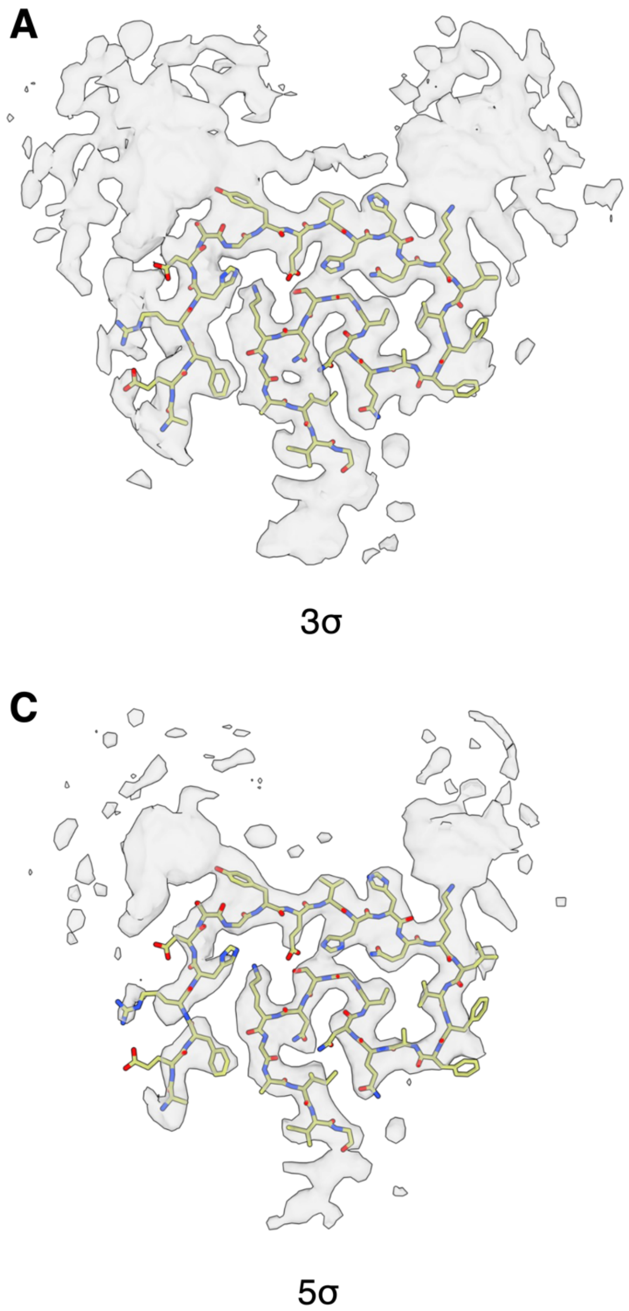

Figure S5: DI3 density map at different thresholds. At (A) 3σ, (B) 4σ, (C) 5σ, and (D) 6σ.

A

	Cfree	Cwork		1 – 32	
1	0.21486	0.83129	D	AEFRHDSGYEVHHQKLVFFAEDVGSNKGAIIG	LMVGGVVIA
2	0.20279	0.788	DAE	FRHDSGYEVHHQKLVFFAEDVGSNKGAIIGLM	VGGVVIA
3	0.19498	0.79647	DAEFRHD	SGYEVHHQKLVFFAEDVGSNKGAIIGLMVGGV	VIA
4	0.18539	0.80618	DAEFRHDSGY	EVHHQKLVFFAEDVGSNKGAIIGLMVGGVVIA	
5	0.17551	0.80869	DAEFRHDSG	YEVHHQKLVFFAEDVGSNKGAIIGLMVGGVVI	A
6	0.16756	0.79798	DAEFRH	DSGYEVHHQKLVFFAEDVGSNKGAIIGLMVGG	VVIA
7	0.16304	0.7844	DAEFRHDS	GYEVHHQKLVFFAEDVGSNKGAIIGLMVGGVV	IA
8	0.15993	0.78889	DA	EFRHDSGYEVHHQKLVFFAEDVGSNKGAIIGL	MVGGVVIA
9	0.15597	0.80193	AIVVGGVM	LGIIAGKNSGVDEAFFVLKQHHVEYGSDHRFE	AD
10	0.15094	0.78938	DAEFR	HDSGYEVHHQKLVFFAEDVGSNKGAIIGLMVG	GVVIA
11	0.1497	0.79756		DAEFRHDSGYEVHHQKLVFFAEDVGSNKGAII	GLMVGGVVIA
12	0.14226	0.7817	AIVV	GGVMLGIIAGKNSGVDEAFFVLKQHHVEYGSD	HRFEAD
13	0.14006	0.7828	AIVVG	GVMLGIIAGKNSGVDEAFFVLKQHHVEYGSDH	RFEAD
14	0.13798	0.79018	AIVVGGVML	GIIAGKNSGVDEAFFVLKQHHVEYGSDHRFEA	D
15	0.13771	0.80256	AIVVGG	VMLGIIAGKNSGVDEAFFVLKQHHVEYGSDHR	FEAD
16	0.13592	0.79658	AIV	VGGVMLGIIAGKNSGVDEAFFVLKQHHVEYGS	DHRFEAD
17	0.13524	0.8013	AI	VVGGVMLGIIAGKNSGVDEAFFVLKQHHVEYG	SDHRFEAD
18	0.13382	0.78705		AIVVGGVMLGIIAGKNSGVDEAFFVLKQHHVE	YGSDHRFEAD
19	0.12439	0.78646	AIVVGGVMLG	IIAGKNSGVDEAFFVLKQHHVEYGSDHRFEAD	
20	0.1177	0.76346	A	IVVGGVMLGIIAGKNSGVDEAFFVLKQHHVEY	GSDHRFEAD
21	0.11127	0.79328	DAEF	RHDSGYEVHHQKLVFFAEDVGSNKGAIIGLMV	GGVVIA
22	0.09261	0.78783	AIVVGGV	MLGIIAGKNSGVDEAFFVLKQHHVEYGSDHRF	EAD

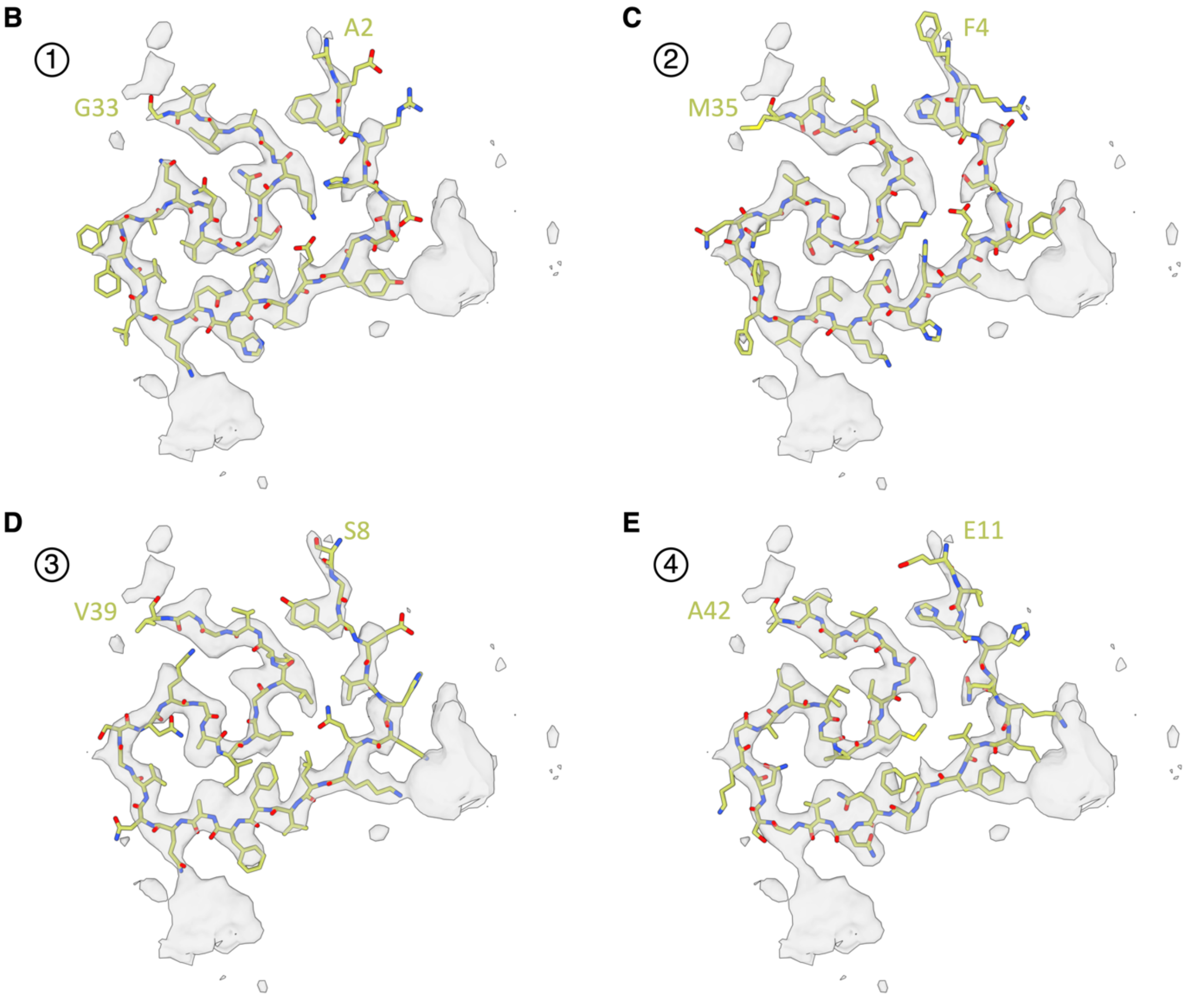

Figure S6: DireX analysis of DI3. (A) The table contains the C_{work} and C_{free} values from the DireX fitting of all possible 22 sequence assignments (black box) in both possible C_{α}-chain directions into the DI3 density maps**.** The C_{free} value was used to rank the results. The N-terminus of the

sequence is coloured in grey, the C-terminus is coloured in green. (B) The most favourable sequence fit. (C)-(E) The 2nd to 4th most favourable sequence fit.

Supplementary Tables

Table S1: Distribution of polymorphs in extracted Aβ fibril samples from different mouse models. The term "unassigned" refers to particles that were originally picked by crYOLO but discarded because they were false positives or of too low quality for high-resolution reconstruction.

Mouse Model	Distribution
APP/PS1	murine type III: 12.2%; unassigned: 87.8%
ARTE10	type II: 13.2%; murine type III: 0.6%; unassigned: 86.2%
tg-SwDI	DI1: 3.6%; DI2: 2.6%; DI3: 0.7%; unassigned: 93.1%
tg-APP$_{\text{Swe}}$	type II: 1.7%; unassigned: 98.3%
APP23	type II: 10%; unassigned: 90%
tg-APP$_{\text{ArcSwe}}$	murine$_{\text{Arc}}$ type I: 4.4%; unassigned: 95.6%
