# Supplementary material for: Cryo-EM of Aβ fibrils from mouse models find tg-APPArcSwe fibrils resemble those found in patients with sporadic Alzheimer’s disease
Source: Nat Neurosci. 2023 Nov 16;26(12):2073–80. doi: 10.1038/s41593-023-01484-4 (PMC10689242; doi:10.1038/s41593-023-01484-4)
Supplement: Supplementary file 2 — Reporting Summary [file 41593_2023_1484_MOESM2_ESM.pdf]

Corresponding author(s): Gunnar Schröder, Lothar Gremer, Dieter Willbold

Last updated by author(s): Sep 6, 2023

Reporting Summary

Nature Portfolio wishes to improve the reproducibility of the work that we publish. This form provides structure for consistency and transparency in reporting. For further information on Nature Portfolio policies, see our Editorial Policies and the Editorial Policy Checklist.

Statistics

For all statistical analyses, confirm that the following items are present in the figure legend, table legend, main text, or Methods section.

n/a	Confirmed	
☐	☒	The exact sample size (n) for each experimental group/condition, given as a discrete number and unit of measurement
☐	☒	A statement on whether measurements were taken from distinct samples or whether the same sample was measured repeatedly
☒	☐	The statistical test(s) used AND whether they are one- or two-sided<br>*Only common tests should be described solely by name; describe more complex techniques in the Methods section.*
☒	☐	A description of all covariates tested
☒	☐	A description of any assumptions or corrections, such as tests of normality and adjustment for multiple comparisons
☒	☐	A full description of the statistical parameters including central tendency (e.g. means) or other basic estimates (e.g. regression coefficient) AND variation (e.g. standard deviation) or associated estimates of uncertainty (e.g. confidence intervals)
☒	☐	For null hypothesis testing, the test statistic (e.g. F, t, r) with confidence intervals, effect sizes, degrees of freedom and P value noted<br>*Give P values as exact values whenever suitable.*
☒	☐	For Bayesian analysis, information on the choice of priors and Markov chain Monte Carlo settings
☒	☐	For hierarchical and complex designs, identification of the appropriate level for tests and full reporting of outcomes
☒	☐	Estimates of effect sizes (e.g. Cohen's d, Pearson's r), indicating how they were calculated

Our web collection on statistics for biologists contains articles on many of the points above.

Software and code

Policy information about availability of computer code

Data collection	EPU 3.2.0, WARP v110Beta, Velox 2.13.0.1138
Data analysis	CTFFIND 4.1; Relion 3.1.0; crYOLO 1.8.4; ChimeraX 1.2.5 and 1.3, Coot 0.8.9.2, ISOLDE 1.3, Phenix 1.20.1, VISDEM 1.0, SCWRL 4.0, CNS1.3, DireX 0.7.1

For manuscripts utilizing custom algorithms or software that are central to the research but not yet described in published literature, software must be made available to editors and reviewers. We strongly encourage code deposition in a community repository (e.g. GitHub). See the Nature Portfolio guidelines for submitting code & software for further information.

Data

Policy information about availability of data

All manuscripts must include a data availability statement. This statement should provide the following information, where applicable:

- Accession codes, unique identifiers, or web links for publicly available datasets
- A description of any restrictions on data availability
- For clinical datasets or third party data, please ensure that the statement adheres to our policy

Cryo-EM maps have been deposited to the Electron Microscopy Data Bank (EMDB) and to the Protein Data Bank (PDB) under the following accession numbers: EMD-16944 (PDB ID: 8OL3) for murine type III Aβ42 fibrils from APP/PS1, EMD-16960 (PDB ID: 8OLO) for murine type III Aβ40 fibrils from ARTE10, EMD-16949 (PDB

ID: 8OL5) for murine type II Aβ42 fibrils from ARTE10, EMD-16959 (PDB ID: 8OLN) for DI1 Aβ fibrils from tg-SwDI, EMD-16957 (PDB ID: 8OLG) for DI2 Aβ fibrils from tg-SwDI, EMD-16961 (PDB ID: 8OLQ) for DI3 Aβ fibrils from tg-SwDI, EMD-16952 (PDB ID: 8OL6) for murine type II Aβ42 fibrils from tg-APPSwe, EMD-16942 (PDB ID: 8OL2) for murine type II Aβ42 fibrils from APP23, and EMD-16953 (PDB ID: 8OL7) for murineArc type I Aβ40 fibrils from tg-APPArcSwe. In addition the raw multi-frame micrographs for the tg-SwDI dataset were deposited to the EMPIAR databank with accession number EMPIAR-11680.

Research involving human participants, their data, or biological material

Policy information about studies with human participants or human data. See also policy information about sex, gender (identity/presentation), and sexual orientation and race, ethnicity and racism.

Reporting on sex and gender	no human material was used.
Reporting on race, ethnicity, or other socially relevant groupings	no human material was used.
Population characteristics	no human material was used.
Recruitment	no human material was used.
Ethics oversight	no human material was used.

Note that full information on the approval of the study protocol must also be provided in the manuscript.

Field-specific reporting

Please select the one below that is the best fit for your research. If you are not sure, read the appropriate sections before making your selection.

☒ Life sciences ☐ Behavioural & social sciences ☐ Ecological, evolutionary & environmental sciences

For a reference copy of the document with all sections, see nature.com/documents/nr-reporting-summary-flat.pdf

Life sciences study design

All studies must disclose on these points even when the disclosure is negative.

Sample size	APP/PS1 (APPswe/PSEN1dE) (heterozygous; n=4 (male = 3; female = 1)); ARTE10 (homozygous; n= 1 (female); Tg-SwDI mice (heterozygous; n = 4 (all male)); APP23 mice (heterozygous; n= 2 (all male)); Tg-APPArcSwe (heterozygous; n= 1 (male)); tg-APPSwe (heterozygous; n= 2 (all male)); No statistical methods were used to pre-determine sample sizes but sample size is limited by the availability of mouse brain material.
Data exclusions	During image processing particles were discarded to obtain high-resolution reconstructions, which is part of the standard image classification procedure. It is possible that not all polymorphs present in the data could be identified and reconstructed. Details of the polymorph distribution and image processing are given in Tables S2 and S3.
Replication	For each mouse model only one dataset was collected. The number of micrographs collected was determined by available EM time and the number of fibrils on the grids as well as the amount of contamination. The amount of data collected (the number of micrographs) was sufficient to generate high-resolution densities at the reported resolutions. The number of datasets collected for each mouse model was limited by the availability of mouse brain material.
Randomization	Randomization was only used for the FSC analysis as shown in Figure S4, where the dataset was divided into two random halves based on a standard approach in RELION 3.1.
Blinding	Blinding was not performed, we think blinding was not relevant to our study as the risk of bias is negligible in this case. In general, blinding is not used when studying molecular structures using cryo-EM.

Reporting for specific materials, systems and methods

We require information from authors about some types of materials, experimental systems and methods used in many studies. Here, indicate whether each material, system or method listed is relevant to your study. If you are not sure if a list item applies to your research, read the appropriate section before selecting a response.

Materials & experimental systems

n/a	Involved in the study
☐	☒ Antibodies
☒	☐ Eukaryotic cell lines
☒	☐ Palaeontology and archaeology
☐	☒ Animals and other organisms
☒	☐ Clinical data
☒	☐ Dual use research of concern
☒	☐ Plants

Methods

n/a	Involved in the study
☒	☐ ChIP-seq
☒	☐ Flow cytometry
☒	☐ MRI-based neuroimaging

Antibodies

Antibodies used

6E10 (BioLegend, Alexa Fluor 594 anti-β-Amyloid, catalog no: 803018, #B309351); 4G8 (BioLegend, catalog no: 800703, #B239200). 6E10 and 4G8 were both diluted 1:500 in TBST with 1% bovine serum albumin (BSA); Nab228 (Sigma-Aldrich, catalog no.: A8354, #0000121536, diluted 1:1000); Goat Anti-Mouse IgG Fc (6nm Gold) preabsorbed (Abcam, catalog no.: ab105285, #GR3386634-1)

Validation

The following target validation statements were provided by the manufacturers: The 6E10 antibody is reactive to amino acid residue 1-16 of beta amyloid. The epitope lies within amino acids 3-8 of beta amyloid (EFRHDS). The 4G8 antibody is reactive to amino acid residues 17-24 of ß amyloid. The epitope lies within amino acids 18-22 of ß amyloid (VFFAE). 4G8 ß-amyloid antibody reacts to abnormally processed isoforms, as well as precursor forms. The Nab228 antibody recognizes human β-amyloid peptide, full-length amyloid precursor protein (APP), soluble-APP (sAPPβ′ and sAPPα), C99 cleavage form, and Aβ (1-40/42), but not soluble-APP form sAPPβ.

Animals and other research organisms

Policy information about studies involving animals; ARRIVE guidelines recommended for reporting animal research, and Sex and Gender in Research

Laboratory animals

APP/PS1 (APPswe/PSEN1dE) (heterozygous; n=4 (male = 3; female = 1); age: 27–33 months old); ARTE10 (homozygous; n= 1 (female); age= 24 months old); Tg-SwDI mice (heterozygous; n = 4 (all male); age: 26–29 months old); APP23 mice (heterozygous; n= 2 (all male); age= 21 months old); Tg-APPArcSwe (heterozygous; n= 1 (male); age= 18 months old); tg-APPSwe (heterozygous; n= 2 (all male); age= 22 months old)

Wild animals

No wild animals were used in this study

Reporting on sex

male=12, female=2. The choice of mouse sample was limited by availability of brain material.

Field-collected samples

No field collected samples were used in the study.

Ethics oversight

APP/PS1, ARTE10, tg-SwDI, APP23 experiments were performed in accordance with the German Law on the protection of animals (TierSchG §§7–9). Breeding of APP/PS1 mice was approved by a local ethics committee [Landesamt für Natur, Umwelt und Verbraucherschutz Nordrhein-Westfalen (LANUV), North Rhine-Westphalia, Germany, Az: 84-02.04.2014.362] before start of the study. APP/PS1 and tg-SwDI mice were (and can be) purchased by the Jackson Lab (JAX MMRRC Stock# 034829 or JAX MMRRC Stock# 034843).The tg-APPArcSwe and tg-APPSwe mice were bred under the ethical permit 5.8.18-20401/20 approved by the Uppsala County Animal Ethics board. All mice were kept and bred under controlled conditions with 12/12 h light/dark cycle, 54% humidity, a temperature of 22°C as well as food and water ad libitum.

Note that full information on the approval of the study protocol must also be provided in the manuscript.
